# A review of piezoelectric MEMS sensors and actuators for gas detection application

**DOI:** 10.1186/s11671-023-03779-8

**Published:** 2023-02-27

**Authors:** Saeed S. Ba Hashwan, Mohd Haris Md. Khir, Illani Mohd Nawi, Mohamad Radzi Ahmad, Mehwish Hanif, Furqan Zahoor, Y. Al-Douri, Abdullah Saleh Algamili, Usman Isyaku Bature, Sami Sultan Alabsi, Mohammed O. Ba Sabbea, Muhammad Junaid

**Affiliations:** 1 Department of Electrical and Electronic Engineering, Universiti Teknologi PETRONAS, 32610 Seri Iskandar, Malaysia; 2 Nanotechnology and Catalysis Research Centre (NANOCAT), University of Malaya, Kuala Lumpur, Malaysia; 3 Department of Mechanical Engineering, Faculty of Engineering, Piri Reis University, Eflatun Sk. No: 8, 34940 Tuzla, Istanbul, Turkey; 4 Department of Applied Science and Astronomy, College of Sciences, University of Sharjah, Sharjah, United Arab Emirates; 5 Department of Electrical and Computer Engineering, University of Waterloo, Waterloo, ON N2L 3G1 Canada; 6 Department of Electronic Engineering, Balochistan University of Information Technology, Engineering and Management Sciences, Quetta, 87300 Pakistan

**Keywords:** Microelectromechanical system (MEMS), Gas sensors, Piezoelectric actuators, Sensing principle, Microcantilever, QCM, SAW, BAW, PMUT, FBAR, Sensing layers

## Abstract

Piezoelectric microelectromechanical system (piezo-MEMS)-based mass sensors including the piezoelectric microcantilevers, surface acoustic waves (SAW), quartz crystal microbalance (QCM), piezoelectric micromachined ultrasonic transducer (PMUT), and film bulk acoustic wave resonators (FBAR) are highlighted as suitable candidates for highly sensitive gas detection application. This paper presents the piezo-MEMS gas sensors’ characteristics such as their miniaturized structure, the capability of integration with readout circuit, and fabrication feasibility using multiuser technologies. The development of the piezoelectric MEMS gas sensors is investigated for the application of low-level concentration gas molecules detection. In this work, the various types of gas sensors based on piezoelectricity are investigated extensively including their operating principle, besides their material parameters as well as the critical design parameters, the device structures, and their sensing materials including the polymers, carbon, metal–organic framework, and graphene.

## Introduction

Microelectromechanical systems (MEMS) originally referred to the integration of the mechanical and electrical components at the microscale and nanoscale dimensions. The main purpose and function of the MEMS are to collect physical and chemical information such as pressure, temperature, chemical and gases molecules from the surrounding environment and deliver this information in a more suitable form to human senses [1]. Undoubtedly, the task of gathering and transforming information is usually performed by sophisticated technical systems. However, MEMS devices are capable to perform these tasks despite their small sizes [2]. In addition, MEMS can be defined as miniaturized mechanical and electromechanical elements that are made through microfabrication techniques with dimensions varying from below one micron in the smallest elements all the way to several millimeters [2–7]. MEMS devices have been designed in several structural varying from simple structural with an element that does not perform any movement to extremely complex electromechanical system that contained multiple elements that performed sophisticated action and movement under the control of integrated microelectronic circuits [8].

The well-addressed components of the MEMS devices are the microsensors and microactuators, also known as “transducers,” which are defined as the elements that perform the task of converting the energy or power from one domain to other domains [9]. For instance, the sensors can convert a measured physical signal into an electrical signal, whereas the actuators can convert the electrical signals into mechanical signals just to move themselves or any other components from one position into another state inside the system. In particular, the sensors are the devices that detect and monitor events or changes in the environment such as gas, chemical, pressure, temperature, vibration, and flow. On the other hand, the actuator transducer is the part of the system that helps to achieve physical/mechanical movement after receiving energy in the form of electrical or other forms of energy. There are various actuators such as pneumatic actuators [10] where their input is air, as well as piezoelectric actuators [11] where their inputs are current or voltage, the micro-valves for controlling the gas and liquid flows, as well as the micro-pumps for fluids pressures [12] that have been used in medical devices and many more. In fact, the output in the actuators is always in the mechanical form of energy [13]. In simple words, the sensing process can be defined as energy transduction that provides us with understanding signals or recognition of unknown actions, whereas the actuation process can be classified as the energy conversion that produces mechanical actions [14, 15].

In addition, MEMS is one of the most promising technologies of the twenty-first century; it has the potential to significantly alter all aspects of our lives and the way we live in the future [16]. MEMS along with the combination of silicon-based microelectronics and micromachining technology has dramatically revolutionized both the industry technologies and consumer products from high-technology machines to tiny elements in smartphones. Scientists believe that the MEMS revolution is going to be the second revolution in micromanufacturing after the semiconductor microfabrication revolution.

The arguable history of MEMS began back on April 1, 1954, when C.S smith [17] from Bell Telephone Lab published in a physical review journal describing the basics of MEMS for the first time which related to the certain stress–strain effects in the silicon and germanium called the piezoresistance. Since then, the researchers have extensively investigated the technologies that have made the transistor and its feasibility to produce sensors and trying to produce electrochemical systems with smaller dimensions [1, 18]. In 1959, a famous talk has been conducted by Richard Feynman entitled “There is plenty of room at the bottom” and he published a summary of the talk later in 1992 [19]. He was interested in exploring how to produce complex motors and machines with multi-functions on a small scale [20]. Richard Feynman and Gordon Moore are only examples of the early scientists who predicted the emerging technologies that could produce tiny microsystems. Currently, new MEMS technologies and applications are being developed every single day globally. Additionally, MEMS are being manufactured using a variety of materials such as semiconductors, biomaterials, nanomaterials, magnetic, piezoelectric, ferroelectric, ceramic, and plastics [11, 21–24]. In addition, MEMS devices can be used in various applications such as sensors, actuators, switches, inertial sensors including (gyroscopes and accelerometers), optical scanners, miniature robots, micro-mirrors, and many more applications are being developed every day [25–33].

In the last few years, MEMS fabrication technology has grown dramatically to the point that tiny devices can be manufactured to be working as actuators and sensors; therefore, they can be found everywhere from wearable devices to automotive equipment [29, 30, 34]. The fabrication techniques used in MEMS production combine the capabilities of the techniques that are utilized in the IC domain with the processes of micromachining such as surface micromachining, bulk micromachining, Lithographie Galvanoformung Abformung (LIGA), high-aspect-ratio micromachining (HARM) to wafer bonding and molding, etc. [30]. MEMS CAP Inc., USA, is one of the famous companies that provides MEMS fabrication facilities for researchers through multiusers MEMS procedures (MUMPs) including several standard fabrication procedures such as MetalMUMPs [35], PolyMUMPs [36], SOIMUMP [37], and PiezoMUMPs [38], whereas there are hundreds of other fabs in the global who are fabricating MEMS devices, just to mention some, including SilTerra [39–41], Infineon [42], CEA-LETI [43, 44], CSEM [45], TSMS [46, 47], and Bosh [48, 49].

Over the above, MEMS technology has been utilized widely in the development of gas and chemical sensors [28]. Moreover, MEMS devices have been used for gas detection including sulfur dioxide (${\textrm{SO}}_{2}$) [50], carbon dioxide (${\textrm{CO}}_{2}$) [51], nitrogen dioxide (${\textrm{NO}}_{2}$) [52], and a few more gases that are constantly released by industry into the environment in the industrialization era. There are various gas sensors utilized in MEMS technologies including capacitive sensing [53], piezoresistive sensing [54, 55], optical sensing [56], and piezoelectric sensing methods [15]. In this review paper, the piezoelectric sensing methods for gas detection applications are presented in detail. There has been significant improvement in the piezoelectric acoustic resonators for gases detection routes such as microcantilever [57, 58], surface acoustic wave (SAW) [59], quartz crystal microbalance (QCM) [60], film bulk acoustic resonator (FBAR) [61], and piezoelectric micromachined ultrasound transducers (PMUT) [62].

Furthermore, nanomaterials that have been used as sensing layers in the gas sensors are playing important roles in the sensor’s structures and functions. The development of sensitive nanomaterials has grown dramatically to enhance and optimize the performance and compatibility of MEMS-based gas sensors. There are various nanomaterials have been developed for sensing toxic and harmful gases. Some of the existing materials are including metal oxide semiconductors (MOS) [63], nanometals particles [64, 65], transition metal dichalcogenides (TMDs) [66], carbon nanotubes and their derivatives [23], graphene and its derivatives [23, 67], and metal–organic frameworks (MOFs) [68], etc., which possess remarkable properties such as high surface to volume ratio, high sensitivity, good reversibility, chemical stability, special chemical bonds, and excellent electrical properties. The combination of highly sensitive nanomaterials and highly precise microfabrication technologies brings a novel solution for gas sensor development. In addition, researchers and scientists have extensively investigated several strategies for enhancing the performance of the gases sensors such as optimizing the device geometry [69], optimizing novel fabrication processes [70], enhancing the resonant frequency as in mass-sensitive sensors [71], and exploring new novel ultrasensitive materials [72, 73]. However, the metal oxide semiconductor sensors are working based on high temperature [74], which not only increase the power consumption but also cause some material defects and safety issues. Therefore, the gas sensors to some extent are vigorously dependent on the novel characteristics of the nanomaterials [75, 76]. In more detail, there are abundant types of nanomaterials that have been developed in various structures and used in the gas sensors such as 0D nanoparticles [77], 0D quantum dots [78], 1D nanowires [79], 1D nanofibers [80], 1D carbon nanotubes [81], 1D nanorods [82], 2D nanosheets [83], 2D honeycomb-like [84] and 3D hierarchical microsphere architectures [85, 86].

Zhu et al. [87] have summarized the future trends of the MEMS device and their application from the 1950s to the current devices. They have summarized various important aspects in the MEMS industries, including the critical microfabrication processes technologies, their operation frequencies, reduction in power consumption, and signal noise. In addition, the future MEMS trends have been addressed such as the wearable MEMS and the adaption of machine learning technologies which help to overcome certain issues in MEMS application [87].

**Fig. 1:**
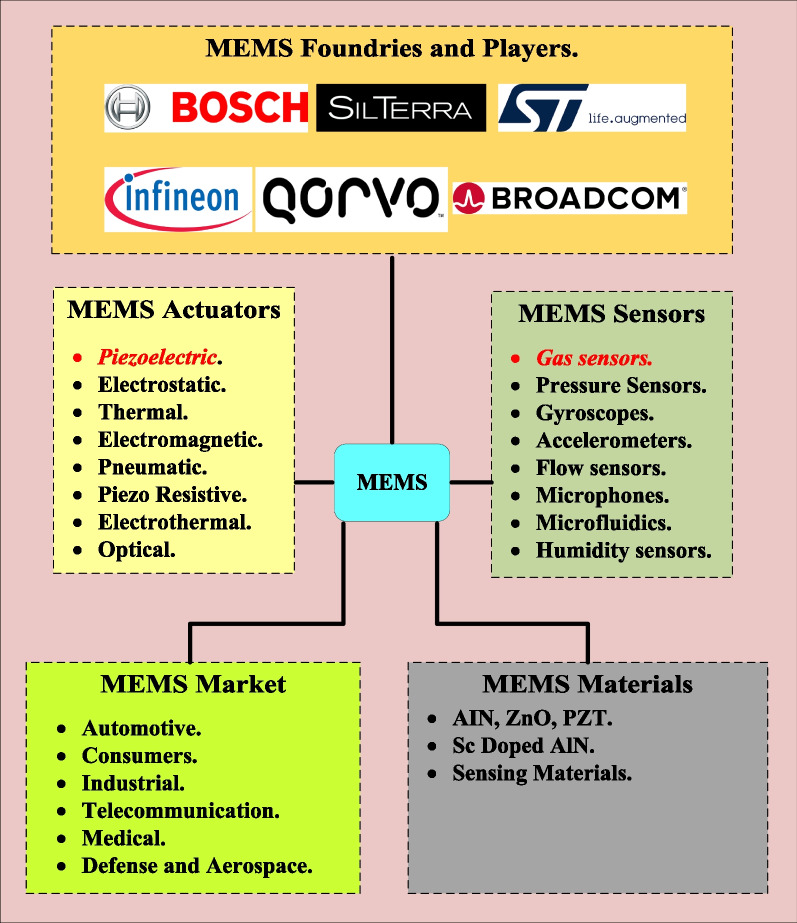
Overview of the MEMS actuators, sensors, famous foundries, piezoelectric materials and the MEMS market

Figure 1 illustrates an overview of the MEMS actuators and sensors as well as highlighted some MEMS materials, the MEMS market, and MEMS famous foundries.

In the next sections, more details about MEMS gas sensors are illustrated including the performance indicators of the gas sensors as in section "Gas sensors key performance indicators", the classifications of the gas sensors as in Section "Classification of gas sensors", as well as the piezoelectric MEMS actuators and sensors transducers as in Section "Piezoelectric MEMS actuators and sensors", and piezoelectric MEMS resonant modes based on the bulk acoustic wave as in Section "Piezoelectric MEMS resonant modes based on bulk acoustic wave" including thickness shear mode as in Section "Thickness shear mode (TSM)", lateral extensional mode as in Section "Lateral extensional mode (LE), contour-mode, or lamb wave mode", thickness extensional mode as in Section "Thickness extensional mode (TE)" and flexural mode as in Section "Flexural mode (Flex) for microcantilever". Furthermore, the piezoelectric MEMS actuators and sensors for gas detection have been demonstrated as in Section "Piezoelectric MEMS actuating and sensing for gas detection". Additionally, more details of the piezoelectric-MEMS devices have been investigated including the microcantilever as in Section "Microcantilever", the QCM as in Section "Quartz crystal microbalance", the SAW as in Section "Surface acoustic wave", as well as the PMUT as in Section "Piezoelectric micromachined ultrasonic transistor (PMUT)", and the FBAR as in Section "Film bulk acoustic resonator".

## Gas sensors key performance indicators

For high-performance gas sensors, there are several indicators or KPIs that the gas sensors must obtain such as high sensitivity, good limit of detection (LOD), excellent selectivity, fast response, repeatability, fast recovery time, or can be called hysteresis or fast reversibility response, low operation temperature, long-term stability, low cost, small size, monolithically, and robust [88]. Furthermore, gas sensors must meet the industrial demands including less consumption of power, easy production, less production cost, easy operation, etc. [89].

In more detail, the device sensitivity is the ratio of the sensor’s output change to the input change, whereas the sensor limit of detection (LOD) can be termed as the sensor’s ability to detect the minimum quantity of the targeted gas; therefore, the sensing materials must show high sensitivity in terms of gas adsorption, or in the form of resistance/capacitance changes due to the small amount of the target reaction with the sensing nanomaterials [90]. Secondly, the selectivity of the gas sensors is defined as the ability of the gas sensors to distinguish and identify a specific gas among various types of gas mixtures [91, 92]. Thirdly, the response time of the sensor, can be termed as the time that the sensor takes to generate a warning signal after the targeted gas molecules reached the sensor surface [93, 94]. The excellent gas sensors are always operated with low response time, in other words, low response time means that the sensor will give a super-fast indication and warning signal [93].

Furthermore, the sensor repeatability is addressed as the sensing materials that are applied to the sensor surface should sense the targeted gas over many detection cycles [95]. In addition, the sensor hysteresis or reversibility is defined as whether the sensor sensing materials could return to the original state after adsorbed the target gas. Moreover, the sensor’s operating temperature should be as minimum as possible to reduce the power consumption and prevent material damage and defects [96]. Additionally, the sensors should present long-term stability which are defined as the ability of the sensor to produce the same output signals for the same input signals for a long interval of time [97, 98].

Ultimately, the final performance and the LOD of the sensor not only depend on the sensor itself, but also depend on the sensor circuit interface, noise-to-signal ratio, and the quantity of frequency change [99].

## Classification of gas sensors

Gas sensors are unique chemical sensors that exhibit variations in at least one of the physical properties of the sensor such as conductance, resistance, absorbance, frequency changes, and temperature variation.

**Fig. 2:**
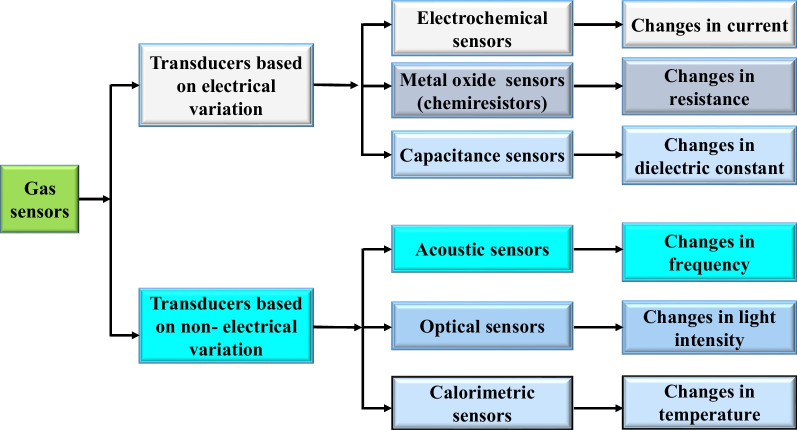
Classification of gas sensors based on transduction methods [97]

There are various types of techniques have been used for gas detection such as electrochemical sensors, metal oxide semiconductors sensors, capacitance sensors, acoustic sensors, optical sensors, and calorimetric sensors. Gas sensors in this research have been classified into two different categories based on electrical and non-electrical properties variation as shown in Fig. 2.

Over the last years, we have witnessed various gas sensors that are developed based on different transduction methods and different sensing materials. Liu et al. [97] have divided gas sensors into two groups as shown in Fig. 2 based on their sensing transduction methods including the methods that are based on the electrical properties variation such as the metal oxide semiconductors sensors, carbon nanotubes sensors and polymer gas sensors, besides the second group which is based on non-electrical properties variation such as the optics based gas sensors, acoustic gas sensors, and calorimetric sensors.

**Table 1:** Summary of gas sensors features based on transduction methods

Sensor types	Mechanism	Features	Refs
Electrochemical	The gases diffused at sensing electrode, then the diffused gas get oxidized or reduced.	-Fast response. High sensitivity and good selectivity. Short operation life.	[328]
Gas chromatography	This method based on separation of gases from the gaseous mixture.	- Expensive. Highly sensitive and excellent selectivity.	[100]
Acoustic	The acoustic detection method is determined by measuring the frequency variation of the acoustic sensor after it absorption/adsorption the gas molecules through the sensing layer.	-Good stability. Good response. Good sensitivity.	[11]
Optical	This detection is based on detection the changes in the optical properties such as colorimetric, reflectance, absorbance, luminescence.	-Long lifetime. Good stability. Good response. Good sensitivity.	[329]
Calorimetric	This detection is based on temperature changes because of the oxidation reaction with the gas molecules	-Less selectivity. Low cost.	[330]

Table 1 summarizes the gas sensors based on their transducer types with brief information about the transduction mechanism and features. Particularly, the table mentioned the common transducers including the electrochemical sensors, gas chromatography, acoustic sensors, optical sensors, as well as the calorimetric sensors. In addition, the gas chromatography and mass spectroscopy (GC-MS) have been used for gas detection and to identify and analyze gaseous molecules with a high capability of generating results rapidly and accurately [100]. However, the GC-MS sensing system requires relatively expensive tools, bulky devices, trained personnel, as well as power-consuming equipment which makes it not suitable for real-time and portable applications [100].

## Piezoelectric MEMS actuators and sensors

Actuators are purely analogue devices that provide the transition of real-world signals into electrical signals for communication with humans [13]. In fact, there is a vast variety of microsensor devices that have been successfully developed and applied in various applications; however, there are only limited applications of the microactuators due to the limitation and insufficient force generated by the small microactuators [101]. Although the most successful microactuators that currently available are dealing with light controlling including optical switches, digital mirror devices, and tunable lenses [102], the piezoelectric and electrostatic microactuators have been shown extremely successful in gas detection applications [103]. Furthermore, thermal actuators have improved significantly and especially in ink-jet printing applications with sharp speed increases thanks to the scaling laws [104]. In addition, extensive development has been contributed to micro-pumps and micro-valves which are always essential for medical implant devices and lab-on-chip applications [105, 106].

In fact, the reduction in MEMS device size is not always preferable due to the huge reduction in the output force of the devices. This can be seen obviously in the electromagnetic microactuators where the size reduction is affecting the output force of the MEMS devices [107]. In addition, the electromagnetic actuators obtained force is a scale to the fourth power of the size; therefore, the electromagnetic actuators are not suitable for small MEMS applications such as gas sensors; however, it is preferable in gigantic projects such as the actuators that are usually used in the satellite [108]. On the other hand, the size issues made the piezoelectric and electrostatic actuations methods as the winners in microsystem applications.

In particular, the piezoelectric actuators are described as the transducers which convert the applied electrical energy into mechanical stress, movement, or strain depending on the type of the piezoelectric materials and the amount of voltage applied [109]. In general, the piezoelectric phenomena are defined as the unique capability or features of some materials -piezoelectric materials- to generate an electrical voltage against any mechanical stress applied on the surface of that materials, and conversely where the piezoelectric crystals can produce mechanical deformation, force, and expand when an electrical voltage is applied [11].

**Fig. 3:**
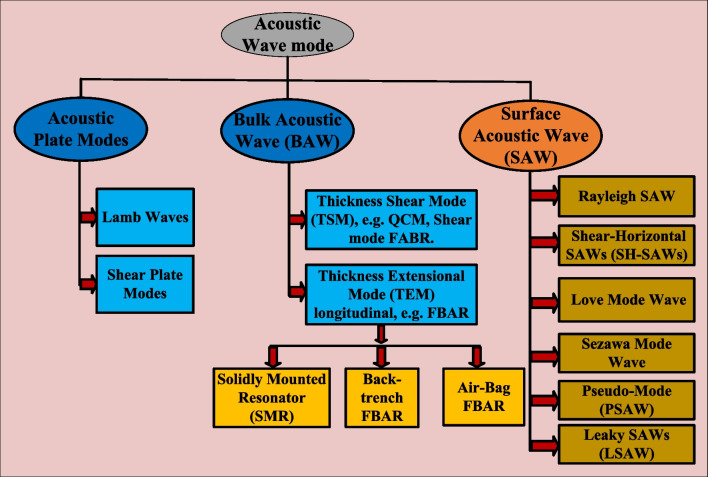
Classification of different wave modes in two categories: surface and bulk acoustic wave (BAW) and surface acoustic waves (SAW)

In more particular, these piezoelectric devices are classified into two main categories based on the acoustic wave propagation mode which are surface and bulk acoustic wave. In surface acoustic wave devices, the acoustic wave propagates parallel to the surface of the piezoelectric substrate [110]. Moreover, in the bulk acoustic devices, the acoustic wave travels and propagates through the piezoelectric crystal in thickness directions [61]. According to this classification, the QCM, FBAR, PMUT, lamb wave, acoustic plate wave (APW), and shear horizontal-APW are among the bulk acoustic devices [111]; on the other hand, the SAW, Rayleigh SAW, SH-SAW, love mode SAW, Sezawa mode wave, pseudo-mode (PSAW), and Leaky SAW are considered among the surface acoustic devices [112]. The thickness shear mode (TSM) as in QCM and the acoustic plate mode resonators are the most widely used BAW devices; besides, the commonly used SAW devices are the flexural plate wave (FPW) and shear horizontal acoustic wave (SH-SAW). Figure 3 illustrates the different types of acoustic wave modes based on the wave propagation method.

## Piezoelectric MEMS resonant modes based on bulk acoustic wave

**Fig. 4:**
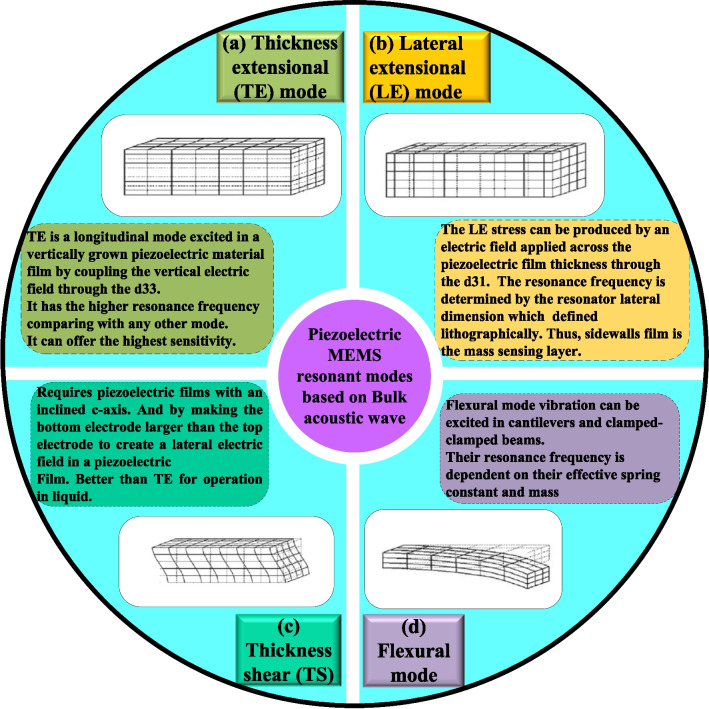
Typical piezoelectric vibration modes based on bulk acoustic waves, (**a**) thickness extensional (TE) mode, (**b**) Lateral extensional (LE) mode, (**c**) Thickness shear (TS), and (**d**) flexural mode [119, 136, 289]

Section "Piezoelectric MEMS resonant modes based on bulk acoustic wave" describes the piezoelectric resonant modes based on the bulk acoustic waves that have been developed and utilized in the MEMS sensors. The BAW piezoelectric modes that have been utilized extensively for chemical and gas sensing applications are the thickness shear mode (TSM) as described in Section "Thickness shear mode (TSM)}, contour-mode (lateral extensional mode) as presented in Section "Lateral extensional mode (LE), contour-mode, or lamb wave mode", longitudinal mode (thickness extensional mode) as illustrated in Section "Thickness extensional mode (TE)" and the flexural mode as discussed in Section "Flexural mode (Flex) for microcantilever". Figure 4 illustrates the summary of the four different types of the vibration modes.

### Thickness shear mode (TSM)

The thickness shear mode (TSM) resonators use shear acoustic vibrations which are transferred to the surface of the device. The TSM devices are considered the most commonly used bulk acoustic wave (BAW) resonators [113]. Quartz crystal microbalance (QCM) and some kind of film bulk acoustic wave resonators are the most commonly used TSM devices for gas sensing; however, there are two different types of the FBAR which are the longitudinal and the shear mode resonators. The main difference between the shear and the longitudinal FBAR mode is in the sputtering process where the shear mode required a certain type of c-axis angles [114]. The scientists discovered that the pure longitudinal wave can be excited at an angle of 0 and 64, while the pure shear wave can be excited at angles of 42 and 90 [114].

Furthermore, it has been found that the sensors with enhanced sensitivity and high performance for liquid application are the shear mode resonators compared with the longitudinal mode [114, 115]. Since the thickness-shear mode radiates less heat into the liquid than a longitudinal wave and does not cause molecules to move perpendicular to the resonator surface, it is thought to be superior to the thickness extensional mode and longitudinal mode for use in a liquid environment [116]. However, the thickness-shear mode has less quality factor and it is not preferable for gas sensing. Additionally, the longitudinal mode is considered the best option for gas detection sensors [117].

In addition, when the piezoelectric thin film is tilted at angles of 34 and 0, respectively, the longitudinal and shear waves exhibit their highest electromechanical coupling coefficients [112, 118].The shear mode in thin film bulk acoustic resonators has been demonstrated to be typically triggered by the deposition of a piezoelectric thin film with an angled c-axis, where the c-axis is not parallel to the thin film plane [119]. To stimulate the shear mode, which causes a shear deformation, a distinct electric field has to be used between the top and bottom sandwiched electrodes. This deformation causes the top and bottom electrode surfaces to move parallel but in opposite directions, creating acoustic waves that travel in the thickness direction [114].There are other methods for generating a thickness shear mode, such as making the bottom electrode bigger than the top electrode, which creates a lateral electric field in the piezoelectric film [120]. The thickness-shear mode’s resonance frequency is dependent on the thickness of the piezoelectric film and the acoustic wave velocity within the piezoelectric materials [121]. Section "Quartz crystal microbalance" describes the thickness-shear mode and its application as gas sensors.

### Lateral extensional mode (LE), contour-mode, or lamb wave mode

The lamb wave resonator, or can be called the contour-mode resonator was first demonstrated in 1973 by Toda [122]. In that experiment, the lamb wave resonator was excited by IDTs on unpolarized PZT ceramic plates. Since then, the Lamb wave resonators were extensively investigated for RF and sensing applications. In addition, the Lamb waves technology was brought up again by Piazza et al., around 2005 basically for RF application [123–125]. Subsequently, the development of the Lamb wave resonators was intensively investigated in various applications including the gas sensors. In fact, the structure of the Lamb wave resonator contained both the SAW and the FBAR structure, it can consist of interdigital transducers and FBAR on the cavity or on the SMA structure. Therefore, it has both advantages from these two technologies. It has the IDTs structure so its frequency can be defined by the lithography processes and its suspended structure of the FBAR enables higher quality factor and larger phase velocity [126]. The contour-mode resonator (CMR) or the Lamb wave mode is the MEMS resonator that operates in the transverse direction, of which the resonant frequency is defined and determined by the in-plane dimensions [125], In this case, however, the resonant frequency is determined by the lateral dimension of the resonator, which can be defined lithographically, rather than the thickness of the piezoelectric material [127]. Therefore, by using this technology different devices can be fabricated in a single chip with different frequencies [128].

**Fig. 5:**
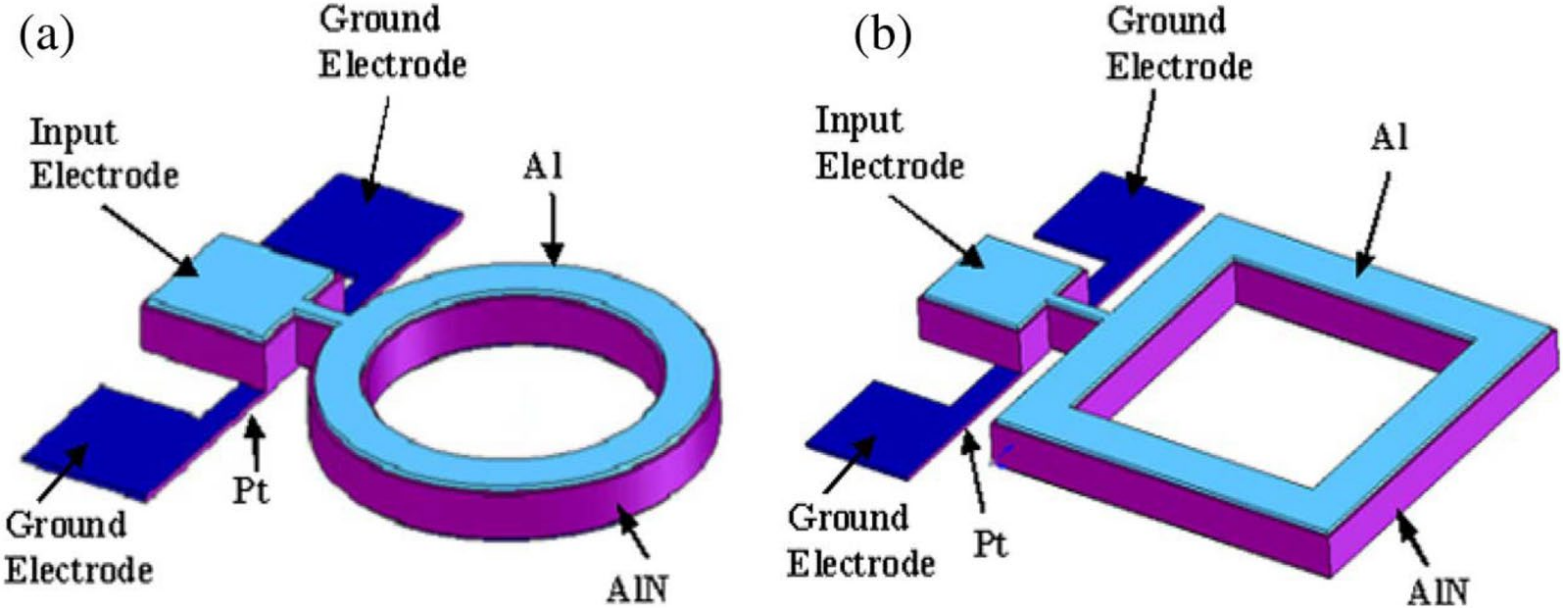
Micromechanical AlN ring-shaped contour-mode resonators: (**a**) One port circular ring and (**b**) One-port square-shaped ring [125]

The working principle of the contour mode is depending on the applied AC signal into the device in the direction perpendicular to the surface of the piezoelectric film. Therefore, the electric field supplied across the thickness of the piezoelectric film through the $d_{31}$ piezoelectric coefficient can result in either the contour mode or lateral extensional mechanical stress [129]. Additionally, the resonator structure vibrates in a dilation-type contour mode as a result of this lateral extensional stress, which also excites a longitudinal wave moving laterally [130]. There are two different approaches that can be used to excite the Lamb wave resonators. The first approach is based on the lateral field excitation (LFE) method and the second approach is based on the interdigital transducers (IDT) [126]. In the contour mode, the mass sensing areas are located at the sidewalls of the piezoelectric film as shown in Fig. 5. Thus, the quality factor in a liquid environment can be higher than in a dry one because relatively little longitudinal wave from the piezoelectric sidewalls is transmitted into the liquid [119].

Lamb wave resonators are preferable for biosensors and chemical sensors which operate in the liquid environment due to the physical separation between the analyte and the transducer surface [119, 131]. Thin film plate acoustic resonators (FPAR) have been presented by Arapan et al. [132], for mass sensitivity through Lamb wave technology. The resonators have been theoretically studied, predicated using the finite element method modal, and experimentally verified [132].

**Fig. 6:**
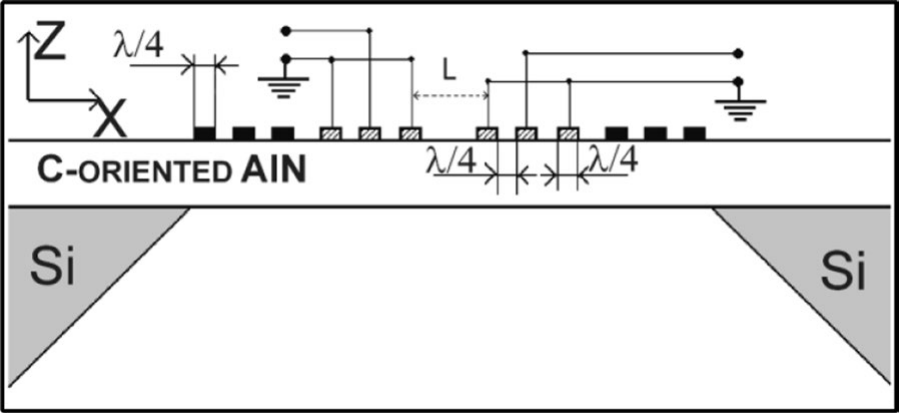
Schematic of 2-port FPAR Lamb wave resonator resonator [132]

Since the Lamb wave resonators have the combined structure of the SAW and the FBAR [133] as presented in Fig. 6; thus, they have the ability to have high resonant frequency and multi-frequency on a single chip, besides high-quality factor, the device can obtain moderate electromechanical coupling coefficient [132]. The phase velocity of the lower-order symmetric Lamb wave mode is up to 10,000 m/s [126]. However, there are some parameters that still need optimization to further reduce the noise in the designed sensors and filters and produce low-lose filters and stable oscillators [133–135]. In addition, the fabrication of these types of resonators is considered not compatible with some common multiuser fabrication technology which is considered one of the obstacles for us to use it in our research.

### Thickness extensional mode (TE)

The thickness extensional mode (TE) is considered the most useful mode for the gas sensor application; however, it is not preferable to be used in a liquid environment due to the high damping caused by the liquid when the sensor immersed in fluid in which the liquid absorbs the acoustic energy [136–138]. In fact, the sensor in the TE mode can be excited by coupling the electric field through the $d_{33}$ piezoelectric coefficient using a vertically grown piezoelectric material such as AIN, PZT, and ZnO. The device should have top and bottom electrodes that sandwich the piezoelectric film and an AC voltage is usually applied on these two electrodes which are required to excite the longitudinal resonance. The TE vibration mode always has higher resonance frequency and wave velocity compared with any other modes; therefore, it has a higher sensitivity for mass sensing applications [119, 139]. More information and explanation about the TE mode are presented in Section "Film bulk acoustic resonator"

### Flexural mode (Flex) for microcantilever

**Table 2:** Resonant frequency equations and their range for the four different modes of the piezoelectric vibration based on bulk acoustic waves

Parameters	Thickness shear (TS) mode	Thickness extensional (TE) mode	Lateral extensional (LE) mode	Flexural (Flex) mode
Resonant frequency equation	$f_{0}=\frac{1}{2d}\sqrt{\frac{c}{\rho }} \quad (1)$	$f_{0}=\frac{1}{2d}\sqrt{\frac{c_{33}}{\rho }} \quad (2)$	$f_{0}=\frac{1}{2W}\sqrt{\frac{c_{31}}{\rho }} \quad (3)$	$f_{0}=\frac{1}{2\pi }\sqrt{\frac{k}{m}} \quad (4)$
Range of $f_{0}$	5–25 MHz (see Table 3)	800–4.8 GHz (see Table 5)	50–160 MHz	0.03–3.3 MHz

The microcantilevers, clamped beams, membrane, and clamped–clamped beams are all vibrated in the flexural mode by utilizing a thin piezoelectric film to one or both sides of the beam’s structure [119]. The flexural mode can be obtained by applying an RF signal across the piezoelectric film, which causes the piezoelectric film to contract and expands depending on the applied frequency. The applying voltage with certain RF causes the entire structure to bend, including the piezoelectric thin film and the attached other materials which form the cantilevers or beams [140]. These piezoelectric beams will be bending and vibrating in flexural mode according to the strength of the applied voltage at the same frequency. If the frequency of the applied voltage is the same as the resonant frequency of the structure, the amplitude of the vibration of the beam will be increased by around Q factor [119]. The cantilevers’ resonant frequency is determined by the spring constant and the mass of the cantilevers. The resonance frequency of the cantilevers will be reduced due to the mass added on its surface after the functionalized layer captured the target molecules. The adsorption of the target imposes some stress changes on the cantilevers’ surface affecting the stiffness of the cantilevers [141]. Section "Microcantilever" presents more details about the microcantilevers and investigated their application, and Table 2 presents the summary of the parameters of the four different piezoelectric vibration modes based on the bulk acoustic waves.

## Piezoelectric MEMS actuating and sensing for gas detection

**Fig. 7:**
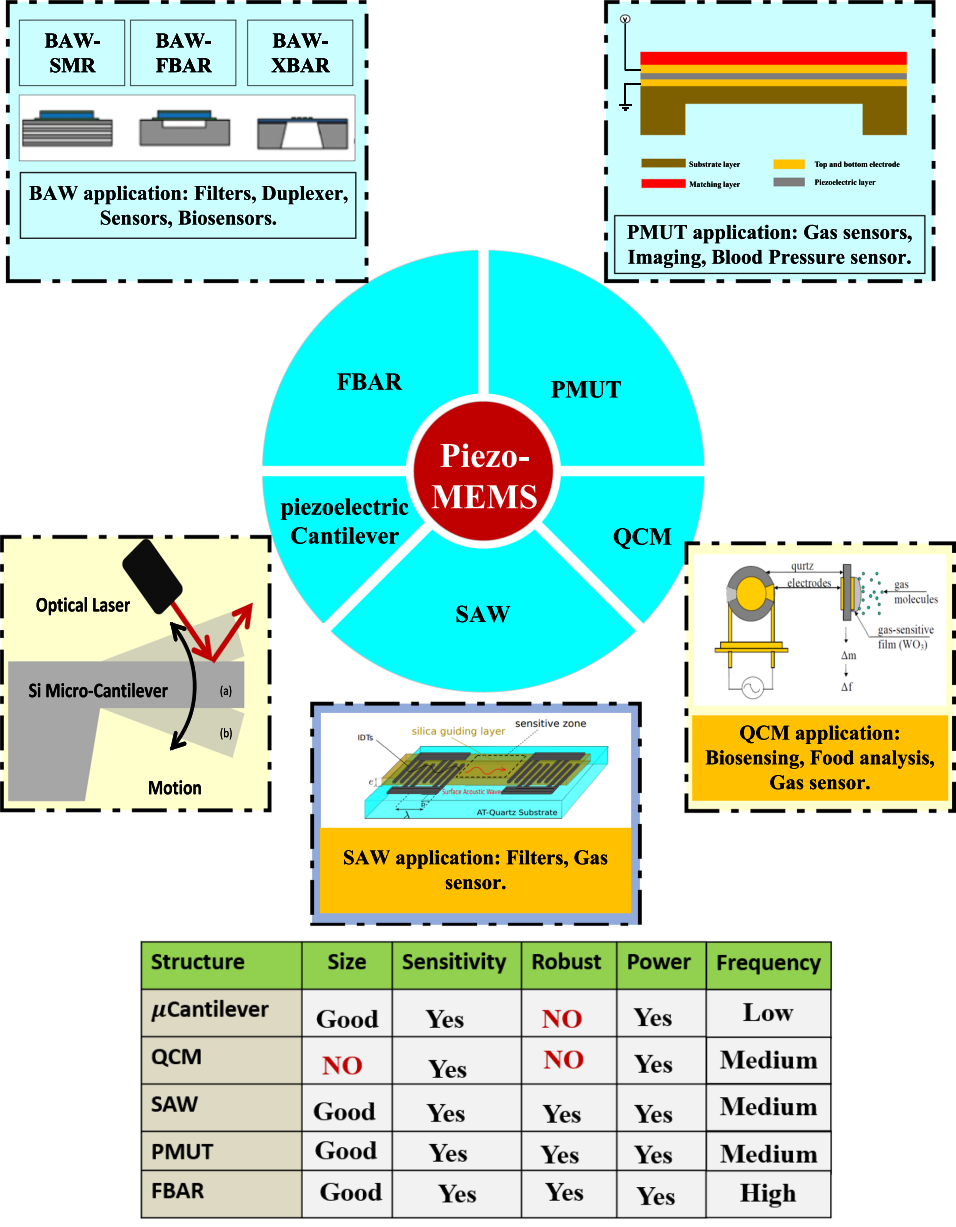
The Schematic for the film bulk acoustic wave, Piezoelectric micromachined ultrasonic transducer, Micro-cantilever, Quartz Crystal microbalance [346], Surface acoustic wave [214]

The piezoelectric MEMS actuators and sensors based on the BAW will be investigated in detail and their structures are presented and summarized in Fig. 7.

Section "Microcantilever" introduces the piezoelectric microcantilever, Section "Quartz crystal microbalance" presents the quartz crystal microbalance, Section "Surface acoustic wave" explains the surface acoustic wave resonators for gas sensors application, Section "Piezoelectric micromachined ultrasonic transistor (PMUT)" introduces the application of the piezoelectric micromachining ultrasonic transducers for gas sensors application, and Section "Film bulk acoustic resonator" investigates the working principles of the FBAR extensively.

### Microcantilever

**Fig. 8:**
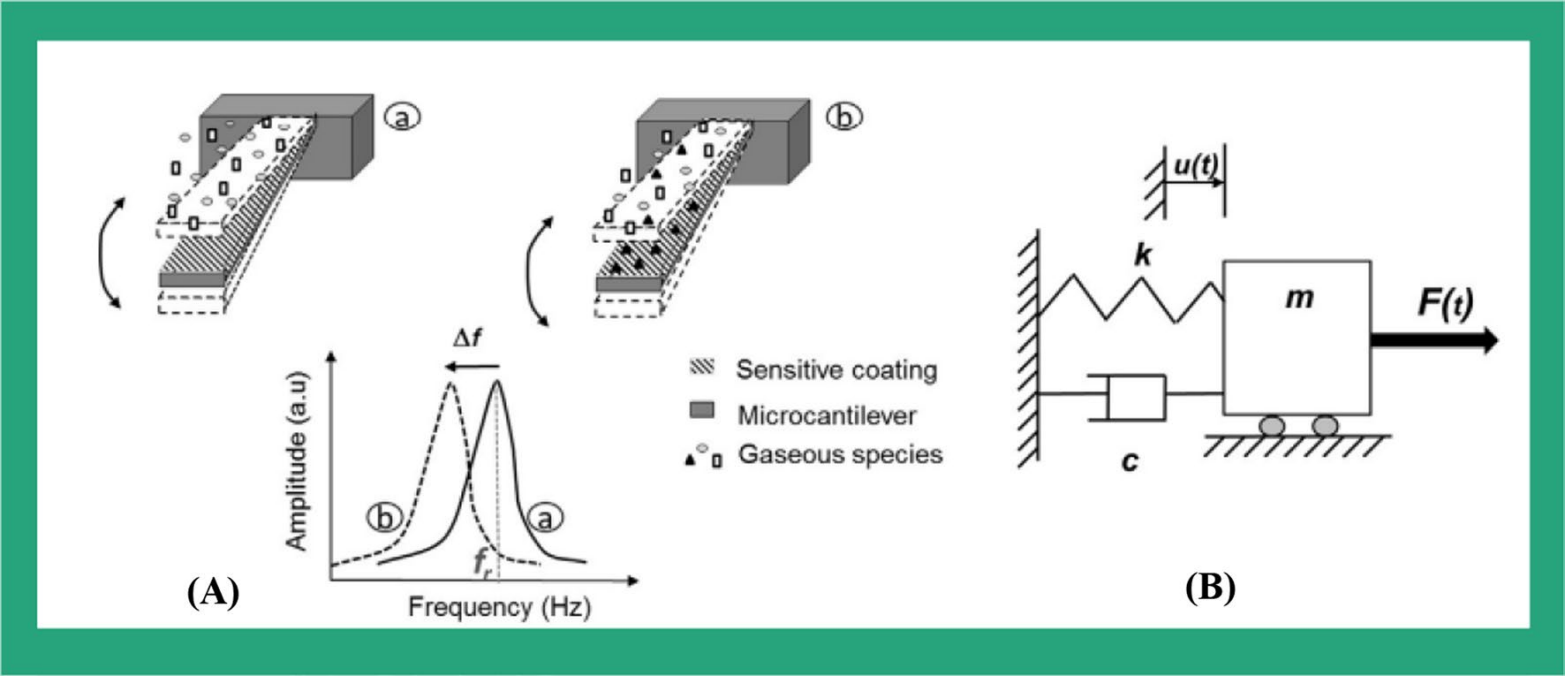
Illustration of microcantilever utilized as chemical sensors. (**A**) presents the microcantilever detection scheme, (**B**) shows the mass-spring-dashpot system equivalent to the vibrating microcantilever [347]

Microcantilevers are the most simplified MEMS-based devices [142], which have been used in various applications such as physical, chemical, and biological sensing. They have been used for blood glucose monitoring [143], gas molecules detection, and disease screening [144, 145]. Furthermore, microcantilevers have been utilized in atomic force microscopy (AFM) for the topography imaging of the surface for almost the last four decades [146, 147]. Additionally, the microcantilever beams have demonstrated their capability as highly sensitive, fast-responding sensors with miniaturized size and low fabrication cost which have been used for various applications. Theoretically, the microcantilever MEMS sensors are responding by bending their structure as shown in Fig. 8 because of the mass changes induced by the adsorption of the analyte molecules on the surface of the cantilevers which lead to a shift in their resonance frequency [142, 148].

The cantilevers have been used in different environmental media such as gaseous, liquid, or vacuum environments [149–151]. The molecules adsorbed on a microcantilever surface can cause vibrations frequency changes and microcantilever deflection [152, 153]. The changes in the vibration frequency can be used for measuring various parameters such as viscosity, density, and flow rate. The deflection is usually proportional to the analyte concentration. Research on resonant microcantilever sensors has focused on enhancing and improving their mass sensitivity by several methods including introducing new material with unique properties, scaling down, or modifying their structural configuration [154, 155].

The measurement of the variation in resonant frequency or the deflection of the silicon beams induced by the adsorption reaction was already described in the literature back in 1968 by Wilfinger et al. [156], who introduced a large silicon cantilever with structures of $50\times 30\times 8$ mm. The proposed devices consisted of a silicon cantilever which is mechanically deflected by electrically induced thermal expansion. Additionally, silicon piezoresistive elements were used as readout elements to detect the cantilever stress and provide an electrical output. In more detail, the device was actuated via Joule heating and the piezoresistors were used to measure the beam deflection.

Since then, the cantilevers have been used extensively in various applications, perhaps the most common application of the cantilevers is the force and displacement sensors in the AFM. The first cantilever for AFM was most probably introduced by Binning et al. [147] back in 1986, who handcrafted the cantilever by cutting thin films of gold foil. Furthermore, the cantilever has been used to actuate by different methods such as electrothermal [157], piezoelectric [158–160], magnetic [161], and electrostatic actuation [162]. After the introduction of the cantilever in 1968 [156], more research has been done to improve and develop the cantilevers to be used as sensors; for instance, Kolesar in 1985 suggested the use of the cantilever structures to be used as electronic nerve agent detectors [163, 164].

Although there are various actuation methods for cantilever vibration that have been used for different applications, this section is only highlighted the piezoelectric cantilevers for chemicals and gaseous molecules detection. Furthermore, the piezoelectric cantilevers are usually actuated by applying an RF signal into the piezoelectric layer using the inverse piezoelectric effect. In the piezoelectric readout method, piezoelectric materials such as AIN, PZT, and ZnO are usually deposited on the cantilever structure. Littrell and Grosh [165] have investigated and developed cantilever-based MEMS using piezoelectric materials for both sensing and actuating.

In addition, Shin et al. [166] have designed, investigated, fabricated, and examined arrays of a piezoelectric microcantilever with various lengths and shapes to optimize their sensitivity and resonance properties. The solgel method was used for PZT piezoelectric layer fabrication on a low-stress SiN layer. The natural resonant frequency of the fabricated microcantilever was shown to be in the range of 16–26 kHz. Furthermore, the same authors (Shin et al. [167]) have fabricated a PZT microcantilever transducer for miniaturized gas sensors to detect gas molecules such as volatile organic compounds. The microcantilever resonance frequency was in the range of 17–29 kHz. The microcantilever surface was coated by polymethyl methacrylate (PMMA) which is well known for its affinity and high sensitivity toward the primary alcohols. The sensors demonstrated obvious changes in the resonance frequency which shifted toward the lower frequency range as the vapor concentration increased. The resonance frequency shift was measured by complex impedance analysis which only uses the electrical signal output from the microcantilever.

Zhou et al. [168] have presented a self-excited piezoelectric microcantilever for Freon gas detection. To develop the microcantilever, theoretical design studies of the device have been done, and the finite element technique has been used to do harmonic analysis on the device. The theoretical and experimental results for the microcantilever’s natural frequency are 1.697 kHz and 1.646 kHz, respectively. The microcantilever has been fabricated successfully using bulk-micromachining techniques and solgel spin coating for the PZT piezoelectric layer. The microcantilever sensor has been coated with zeolite nanomaterial as a sensitive layer and the sensor has been characterized for 12 different concentrations of Freon gas ranging from 10 to 500 ppm.

### Quartz crystal microbalance

**Fig. 9:**
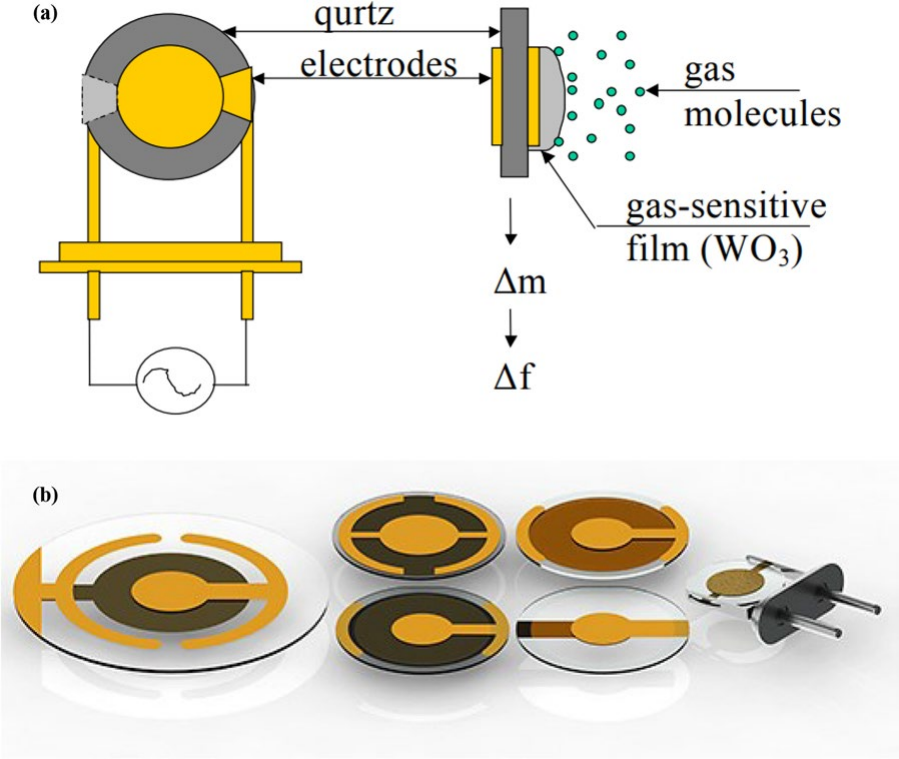
Schematic of quartz crystal microbalance, (**a**) presents the QCM for gas molecules detection, (**b**) shows the different structures of the QCM [346]

Quartz crystal microbalance devices are an extremely sensitive mass balance based on the piezoelectric effect that can measure micrograms to nanograms levels changes in mass per unit area [169]. In QCM devices, the technology is based on the quartz piezoelectric disk material. The QCM devices use the piezoelectric effect of a thin disk of quartz crystal material placed between two metal electrodes on opposite sides of the disk, as shown in Fig. 9. The overlapping parts of the quartz disk with the electrodes define the active sensing surface [170].

The quartz piezoelectric can be made to oscillate at a defined frequency when an alternating electric field is applied via metal electrodes. The oscillation and the vibration motion of the quartz crystal disk piezoelectric materials established a transverse acoustic wave that can propagate across the crystal materials and reflects again into the surface of the crystal. Therefore, the thickness of the quartz piezoelectric plate defines the quantity of the resonant frequency for the QCM devices which range from 5 to 30 MHz [171]. The QCM devices are also known as thickness shear mode (TSM), and they are considered parts of the bulk acoustic wave devices [172]. The resonant frequency of the QCM can be affected by any mass changes occurring in the electrode surface of the device such as the addition or removal of any small amount of gas molecules. Thus, this range of resonant frequency can be monitored in real time to harvest useful information about the reactions or interactions that occurred on the top electrode of the QCM device such as gas molecular interaction with the sensing layer, oxidation, thin film growth, and material corrosion. Hence, the change in the top electrode mass is linearly related to the changes in the resonant frequency of the QCM sensor where this relationship is expressed by the Sauerbrey equation as shown in Eq. (1) [173]:

1 $$\begin{aligned} \Delta f=-\frac{2 f_{0}^{2}}{A \sqrt{p_{q} \mu _{q}}} \Delta m \end{aligned}$$

where $\Delta {f}$ is the change of the resonant frequency in Hz, $f_{0}$ is the resonant frequency of the fundamental mode, $\Delta {m}$ is the mass change in (g), *A* is the piezoelectrically active crystal area which is between the two metal electrodes in ($\textrm{cm}^2$), ${p_{q}}$ representing the quartz crystal density which is equal to 2.648 g/$\textrm{cm}^3$, and the $\mu _{q}$ representing the shear elastic modulus of the quartz which is equal to $2.947 \times 1011$ g$\cdot$(cm$^{-1}$)$\cdot$(s$^{-2}$) [174].

A QCM gas sensor was used for the detection applications of hazardous gases at room temperature by Alev et al. [175]. The surface of the QCM sensor has been deposited by Cu doped with ZnO nanorods (NRs). This sensitive nanomaterial was successfully synthesized from Cu-doped and pristine ZnO nanorods using two-step electrochemical deposition technique, which was optimized in this experiment to get highly ordered ZnO nanorods. The QCM-fabricated gas sensor was tested to detect several gases including ${{\textrm{H}}_{2}}{S}$, ${\textrm{NO}}_{2}$, HCN, isopropyl alcohol, ethyl acetate, xylene, and toluene. In this experiment, the results shown that the process of Cu doping with ZnO nanorods has obviously enhanced the sensor sensitivity at room temperature, especially for ${{\textrm{H}}_{2}}{S}$ and HCN gases. The variation of the Cu doping ZnO concentration has shown that the $1\%$ concentration of the Cu doping ZnO nanorods presented the highest sensor response compared with $3\%$ Cu doped with ZnO, where this increment in the sensor response was justified by the enhancement of the physisorption properties of the NRs surface.

Furthermore, Trajcheva et al. [169] reported the investigation of the QCM gas sensor sensitivity coated with graphene nanoribbons (GNR)/ polymer hybrid nanocomposite for several hazardous gas detection. The GNRs are narrow strips of graphene that are characterized to be in one-dimensional morphology with significantly excellent surface properties, that can offer a huge number of functional groups. The GNR has been mixed with a cheap polymeric material to obtain a highly sensitive nanocomposite. The GNR/polymer nanocomposites have been produced for the first time in this experiment by using the advantages of polymerization in the dispersal media to act as green synthesized material. The interaction between the GNR/polymer nanocomposite was formed by established covalent bonding between the phases of both materials, this type of bonding is responsible for boost of the strong thermal and mechanical in the nanocomposites. This QCM has been deposited with GNR/polymer nanocomposites and exposed to various gases including ${\textrm{NH}}_{3}$, ${{\textrm{N}}_{2}}{O}$, and *CO* in a different amount of concentration range from 70 to 1000 ppm. The developed QCM sensors were characterized at room temperature for three cycles of gas adsorption and desorption and the large number of responses of response in a short time. The QCM sensors have shown excellent performance, especially in the reproducibility of the sensor for the investigated three cycles.

The extraordinary improvement in the sensor performance was attributed to the huge number of functional groups that have been created between the polymer and GNR where these functional groups and the uniqueness of the nanocomposite morphology that offers numerous adsorption agents. The selectivity of the QCM sensor with GNR/polymer nanocomposites was investigated for the three gases and the sensors showed excellent selectivity toward ${\textrm{NH}}_{3}$ gas compared with CO and ${{\textrm{N}}_{2}}_\textrm{O}$. The authors reported that the selectivity of the sensor was due to the interaction between the ${\textrm{NH}}_{3}$ and the nanocomposites by Wan der Waals forces and hydrogen bonding that only formed between the nanocomposites and the ${\textrm{NH}}_{3}$ gas whereas, the other two gases was interacted exclusively by the van der Waals interactions [169].

In addition, QCM sensors’ performance has been enhanced by utilizing highly functionalized reduced graphene oxide (rGO) to detect carbon dioxide at room temperature by Gupta et al. [176]. The thin film rGO was chemically synthesized by chemical reduction in graphene oxide using an Ascorbic acid green agent. In this experiment, the rGO was optimized using three different concentrations of the ascorbic acid reduction agent which are (25, 50, and 100 mg). Three different thin films were prepared and analyzed using several characterizations such as SEM, TEM, XRD, FTIR, XPS, RAMAN, and four-point probe measurement. The rGO thin film with 25 mg of reduction agent showed excellent sensing properties in terms of sensing and time of recovery with enhanced repeatability for ${\textrm{CO}}_{2}$ detection with a variation of 500–50 ppm at room temperature. The QCM gas sensors sensitivity has been investigated for 500 ppm ${\textrm{CO}}_{2}$ gas which shows 50 Hz $\mu {g}$ at room temperature. The response and recovery time of the QCM sensors was reported to be 26 and 10 s, respectively [176].

Furthermore, QCM devices for gas sensors application have been used extensively along with the development of nanomaterials which has enhanced the sensitivity and selectivity of the sensors. A research paper published by Chen et al. [177] demonstrated a unique method for depositing graphene oxide and cuprous oxide (GO/$\textrm{Cu}_2$O) nanocomposites on the surface of the QCM via a layer-by-layer self-assembly technique. This work was used for trimethylamine gas detection with low concentrations under 5 ppm. The response of the QCM sensor has been increased linearly with the gas concentration. The authors reported that the QCM gas sensors presented good selectivity, sensitivity, stability, and reversibility during the 60 days of investigation. The limit of detection (LOD) in this experiment was illustrated to be 230 ppb under room temperature for trimethylamine gas using QCM. The gas detection mechanism using GO/$\textrm{Cu}_2$O nanocomposites was demonstrated as an adsorption–desorption process that was carried out via the interaction called the hydrogen bonding between the carboxyl functional groups on the surface of the GO and the trimethylamine gas molecules. Furthermore, the layer-by-layer self-assembled method has enlarged the surface area of the p-n junction of GO/$\textrm{Cu}_2$O which enhanced the physical adsorption of trimethylamine gas molecules [177].

**Table 3:** Summary for QCM gas sensors

Piezoelectric materials	Device frequency (MHz)	Sensing layer	Target	LOD (ppm)	Frequency shift (Hz/ppm)	Response time (s)	Recovery time (s)	Temp	Year	Ref
AT-CUT	25	PAAM/MWCNTs.	Formaldehyde.	0.5	3	80	100	RT	2021	[331]
AT-CUT	10	Cu-doped ZnO NRs.	H2S	6.6	68	n/g	n/g	RT	2020	[175]
AT-CUT	10	rGO	$\textrm{CO}_2$	50–500	50	26	10	RT	2021	[176]
AT-CUT	10	MOF	$\textrm{CO}_2$	400–500	n/g	n/g	n/g	RT	2018	[179]
AT-CUT	9	(ZIF-8)	$\textrm{CO}_2$	1.000.000	217	10	n/g	RT	2018	[215]
AT-CUT	9	Graphene oxide	Formaldehyde	1.7	22.9	60	n/g	RT	2016	[332]
AT-CUT	9	ZIF	$\textrm{CO}_2$	n/g	n/g	n/g	n/g	RT	2018	[333]
AT-CUT	8	PEI/MWCNTs	Formaldehyde	6	0.82	114	127	RT	2015	[334]
AT-CUT	8	SnO$_{2}$/Polydopamine	Formaldehyde.	0.5-50	12.2	25	38	RT	2021	[335]
AT-CUT	5	polymer/GNR.	NH3	70	106	1800	n/g	RT	2021	[169]
AT-CUT	n/g	GO/Cu$_{2}$O.	Trimethylamine.	5	15.2	20	n/g	RT	2018	[177]

n/g $$=$$ Not Given

Fauzi et al. [178] summarized the recent progress in the development of QCM devices that are coated with graphene materials and graphene composite nanomaterials which are used for gas and humidity-sensing applications. The min review paper mainly focused in the recent advances of the QCM gas and humidity sensors’ performance, especially the characterized of the devices that are coated with pristine graphene, graphene oxide, reduced graphene oxide, and different graphene composite materials such as graphene–metal oxide composite, polymer, chemical, and other carbon-based materials. The report addressed the QCM sensors’ challenges for sensor future development [178]. Table 3 summarizes some of the recently highlighted research for the development of the QCM for gas sensor application.

### Surface acoustic wave

SAW technology produces highly sensitive devices for chemical detection in both gaseous states as well as liquid environments [179]. In 1965 [180] SAW technology was introduced for the first time by White and Voltmer who reported the generation of surface acoustic waves by utilizing interdigitated pair of electrodes called interdigital transducers (IDTs) which were fabricated on a quartz piezoelectric surface and actuated by applying RF voltage [181]. In that contribution, the SAW waves were generated by applying a voltage signal to the fabricated IDTs electrodes on the surface of the devices. Since then, SAW technology has been extensively investigated and developed for wide applications [182].

Wohltjen and Dessy in 1979 [183] used SAW technology for the first time for organic gas detection by coating a sensitive sensing layer on the top surface of the SAW device. This breakthrough attracted the researchers’ attention and a variety of SAW devices has been reported for gas detection. The sensitivity of the SAW sensors highly depends on the sensitivity of the sensing film, coated on the top surface of the device [184]. The sensing layer must possess the capability of adsorbing certain types of gases and does not react with other gases which determined the selectivity of the gas sensors [185].

In addition, the SAW device transducer mainly determines the sensitivity of the SAW gas sensors, while the coated nanomaterial usually determines the selectivity and specificity of the sensor [186]. The SAW sensors technology is considered one type of gravimetric transducers which are relied on Sauerbrey’s classical theory that has been published in 1959 [173]. The Sauerbrey contribution has described the relationship between the weight and the change in the resonant frequency of piezoelectric materials.

Surface acoustic wave devices have been developed in the early stages mainly for certain applications including signal processing [187, 188], resonators, actuators [189], frequency filters [190, 191], and others [192, 193]. However, in the last few decades, there are a significant increase in the SAW for gas, chemical, and biochemical detection application [182]. The SAW sensors have offered several advantages which determine by the piezoelectric transducers such as wired and wireless operation, fast response, ultra-high sensitivity, small size, low cost, and compatibility with modern fabrication technologies [194].

In addition, the SAW sensors can provide more extra advantages which rely upon the proper selection of the coated sensing layer including excellent selectivity, reversibility, stability, linearity, and fast response [195]. The SAW sensors performance determines by some main factors including the piezoelectric substrate, the material of the sensing layer, and the interdigital transducers (IDTs). The SAW gas sensors are intended to address the rapidly increasing need for high-performance gas and chemical sensors in all applications, including pollution monitoring, military, and industrial [196], industries, volatile organic chemicals (VOCs) [110, 197], and detection of other various toxic gases [198, 199].

**Fig. 10:**
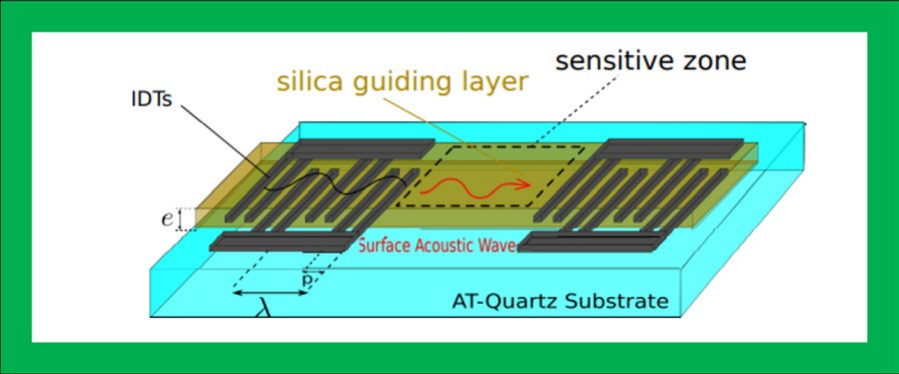
Structure of a surface acoustic wave based on a delay line built on an AT-cut quartz substrate, produced by [214]

The SAW device sensors’ work basis on variations in acoustic wave propagation, that are influenced by interactions between the waves and the environment nearby, such as the target gas or the surface layers. In fact, the SAW acoustic waves depend on the propagation medium characterizes, elastic stiffness, mass density, and electric–dielectric behavior of the piezoelectric materials. There are various types of SAW devices that operated differently. The SAW devices can operate between a few MHz and a few GHz where this frequency is higher than the QCM devices; therefore, the sensitivity of the SAW is considered higher compared with the QCM piezoelectric sensors. A “delay line” SAW sensor is considered fundamental to the SAW devices. It consisted of IDTs deposited on both sides of the piezoelectric substrate where one of them performs as input and the other as output IDT. The IDTs are made from periodic metals that are typically shaped like two combs that cross over from opposite sides. The surface between the two IDT as shown in Fig. 10 is the sensing area where the sensing material is deposited for target detection.

The interaction of the sensing layer and the target gas or chemical in the region between the input and output IDTs causes the time difference between the input and output signals. The length of the sensitive layer as well as the velocity of the SAW material influences how long the output signal is delayed. The presence of the target or analyte in the sensing layer, in particular, changed the acoustic waves’ phase velocity, attenuation, and amplitude.

Hence, these variations in the output electrical signal at the output IDT could yield some useful information [200]. SAW wave propagation in the piezoelectric layer can produce both electrical potential and mechanical deformation [201]. The mass loading on the surface of the sensor and the elastic and viscoelastic are the mechanical deformation caused by the interaction between the sensing layer and the targeted analytes [59]. In addition, these effects are called the acoustoelectric effects which are the effects that result from the interaction between the presented targeted analyte in the sensing layer and the electrical field associated with the SAW waves [202].

Moreover, there are basically three common modes of SAWs devices that are extensively utilized for gases and chemicals detection, namely Rayleigh wave mode [203], Lamb wave mode [204], and shear horizontal wave mode (SH-SAWs) [205]. In addition, most of the reported SAW gas and microfluidic sensors are based on the Rayleigh waves mode [206, 207], whereas the shear horizontal and Lamb wave SAWs are only suitable for gas sensors, but they are not able to perform the detection in the microfluidic or liquid-based sensing environment due to the fact that the wave propagation in the shear horizontal mode and its displacement is only parallel with the substrate surface, which inhibits the wave vibration into the liquid on the surface [181]. The Rayleigh waves are type of surface waves that travel near the surface of solids in both longitudinal and transverse motions [208]. In Rayleigh wave mode, the SAW propagates at the speed of sound on the crystal. However, the amplitude of these waves decreases exponentially with the increase in the distance from the solid’s surface. The Rayleigh waves were predicted by Lord Rayleigh in 1885 [206], after whom they were named. In more detail, the Rayleigh SAW sensors are creating an out-of-plane elliptically polarized surface wave caused by the acoustic energy near the piezoelectric substrate surface [209]. The resonant frequency of the SAW devices [210] is calculated by Eq. (2):

2 $$\begin{aligned} f=\frac{v}{l} \end{aligned}$$

where *v* is the velocity of the wave for the certain substrate material, and the *l* is the wavelength. In fact, the perturbation theory describes the changes in the resonant frequency which will be affected by the mass changes caused by the gas adsorption [211]. Furthermore, the SAW resonant frequency changes that occurred after the gas or analyte absorbed by the coated layer can be expressed by Eq. (3) [212, 213], where the absorbed gas considered as non-piezoelectric, non-conductor, with a density of p, and a thickness h. Equation (3) can be expressed as:

3 $$\begin{aligned} \Delta f=\left( k_{1}+k_{2}\right) f_{0}^{2} \rho h-k_{2} f_{0}^{2} \rho h \frac{4 \mu }{v_{0}^{2}}\left( \frac{\uplambda +\mu }{\uplambda +2 \mu }\right) \end{aligned}$$

where $f_{0}$ is the unperturbed resonant frequency of the SAW oscillator, which is determined by Eq. (2), and it is affected by the propagation velocity of the SAW and the number of the comb fingers that fabricated on the surface of the piezoelectric substrate; $k_{1}$, and $k_{2}$ are the coupling constants which can be determined by the SAW device different displacement components; $v_{0}$ is the unperturbed velocity of the SAW waves in the piezoelectric substrate; $\mu {}$ and $\uplambda$ are the shear modulus and the Lame constant of the layer that have been generated after the gas adsorption. However, this formed layer that has been created by the adsorbed or absorbed gas is very thin layer which made the second part of the equation close to zero. Therefore, the second term of the equation depends on the acoustic wave coupled into the layer whereas, the first part of the equation will be remained to be calculated which represents the SAW resonant frequency changes caused by the mass loading on the surface of the SAW. Therefore, Eq. (3) can be simplified as expressed Eq. (4):

4 $$\begin{aligned} \Delta f=\left( k_{1}+k_{2}\right) f_{0}^{2} p h \end{aligned}$$

where the *ph* is the new density of the layer that has been formed after the gas adsorbed [212, 213]. These equations have been developed theoretically and proven experimentally by Wohltjen, who investigated in detail the relationship between the interaction of the vapor molecules and polymeric coating films on the surface of the SAW device [212]. Furthermore, Djoumi et al. [214] developed a real-time mass sensor using SAW for PM10 and PM2.5 mass concentration measurement. They produced SAW sensors with a working frequency of 125 MHz based on love waves delay lines mode where the waves propagate on AT-cut quartz substrate as presented in Fig. 10

Recently, Palla-Papavlu et al. [186] have published a review paper that presented the latest progress development in the sensing layer nanomaterial including polymers, and functionalized carbonaceous materials, organic salts, and self-assembled monolayers for SAW sensors. The survey paper reported the synthesis processes of the functionalized CNTs and graphene that have been used to enhance the sensitivity of the SAW and other acoustic sensors. Furthermore, the sensing layer coating techniques and methods have been illustrated including physical, chemical, spray coating, ink-jet printing, and other surface modification methods. The authors have compared and highlighted some of the best routes for the enhancement of the acoustic sensors’ performance that is used for dangerous compound detection. In fact, there are many effects have been focused to enhancing the performance of the SAW sensors by either improving the current nanomaterials sensitivity through the modification of their surface morphology and attachment of functional groups that will be binding with the analyte or by synthesis of entirely new sensitive materials [186].

In addition, Jagannath Devkota et al. [215] have designed and fabricated SAW delay line sensors for ${\textrm{CO}}_{2}$ and methane detection at the ambient condition at operating frequency of 436 MHz. The sensitivity of the SAW sensors was enhanced by directly coated zeolitic imidazole framework-8 (ZIF-8) metal–organic framework (MOF) on the surface of the SAW devices. The fabricated SAW sensors were tested for several gases detection, and the devices were able to detect the changes in the concentration of ${\textrm{CO}}_{2}$ and ${\textrm{NH}}_{4}$; however, the sensors’ sensitivity toward ${\textrm{CO}}_{2}$ was much higher compared to ${\textrm{NH}}_{4}$, which was due to the ${\textrm{CO}}_{2}$ higher adsorption potential and their heavier molecular weight.

The SAW gas sensors have shown full reversibility and repeatability which were confirmation of the physisorption of the gases into the MOF indicating the physical bonding of the gases molecular with the surface of the MOF which provides high stability of the sensors. This research confirmed the potential and capability of the ZIF-8 in adsorbing carbon dioxide gas molecules. Furthermore, the authors have published another research paper [216] for wireless and passive SAWs gas sensors using the same sensing layer which was a nanoporous metal–organic framework, specifically the ZIF-8 sensing film for carbon dioxide and methane gas detection at ambient conditions; however, the sensitivity of the reflective delay line SAW gas sensor was enhanced by increasing the resonant frequency from 436 MHz (8 $\mu {m}$ periodicity) to 860 MHz (4 $\mu {m}$ periodicity) [215].

The increase in the operating frequency has enhanced the sensor sensitivity at least four times and the detection limit of the higher frequency devices was estimated to be 0.91 vol%; thereby, the enhancement in the sensor sensitivity by higher frequency devices is explained and confirmed that the acoustic devices are frequency dependent. In this published paper, the design, fabrication, characterization, and parameters classification [216].

Furthermore, the sensitivity, stability, and selectivity of SAW gas sensors were enhanced by the integration of treated lead sulfide (PbS) colloidal quantum dots (CQDs) into the surface of the SAW devices [217]. In that research, the authors investigated the utilization of nanomaterials for SAW gas sensors’ performance enhancement for ${\textrm{NO}}_{2}$ detection at room temperature. The SAW sensors were coated with untreated PbS CQDs which were directly deposited on the delay line SAW devices using spin coating techniques followed by chemical treatment. The experiments illustrated the responses, recovery time and the frequency shift of the SAW sensors using the treated and untreated PbS CQDs nanomaterials. The results were shown that the sensors with untreated nanomaterials shown response and recovery time of 487 s and 302 s with a negative frequency shift of 2.2 kHz.

In contrast, the treated nanomaterials presented dramatic improvement in the sensitivity, selectivity, stability, response, and recovery time at room temperature with a sharp increment in the frequency shifts. In particular, the sensor response and recovery time were reported to be 45 s and 58 s with positive frequency shifts of 9.8 kHz, respectively. The improvement in the treated nanomaterials might be caused by the trapping of the ${\textrm{NO}}_{2}$ molecules into the porous film which increases the film stiffness [217].

Tang et al. [218] have reported SAWs gas sensors for ${\textrm{NH}}_{3}$ gas detection using several sensing layers such as pristine ${\textrm{SiO}}_{2}$, ${\textrm{TiO}}_{2}$, and composite ${\mathrm{SiO-TiO}}_{2}$ films. The thickness of the sensing layers was 200 nm and coated on the surface of quartz acoustic wave sensors using solgel and spin coating techniques. The performance and mechanism of the SAW sensors were systematically investigated. The experiments had shown that the sensors made of ${\textrm{TiO}}_{2}$ and ${\textrm{SiO}}_{2}-{\textrm{TiO}}_{2}$ films exhibited positive frequency shifts toward ${\textrm{NH}}_{3}$ whereas only ${\textrm{SiO}}_{2}$ sensing layer presented a negative frequency shift toward the gas. The authors illustrated that the negative frequency shifts were mainly caused by the mass increase in ${\textrm{NH}}_{3}$ gas into the surface of the SAW sensors.

In contrast, the positive frequency shift was basically associated with the hydroxyl groups (-OH) condensation on the sensing layer film due to the ${\textrm{NH}}_{3}$ exposure; thereby, this reaction is making the film more stiffer and lighter [218]. The fabricated SAW sensors’ performance was characterized under the effect of humidity, and it has been demonstrated that the humidity played a significant factor in the coated SAW sensors’ performance. Additionally, studies in the literature exhibited that the performance of the SAW gas sensors was dramatically enhanced due to the utilization of highly sensitive thin films as it has been proven in this research [218] that the ${\textrm{SiO}}_{2}-{\textrm{TiO}}_{2}$ thin film had increased the sensitivity of the SAW gas sensors for ${\textrm{NH}}_{3}$ gas to lower concentration (1 ppm) with a frequency shift of 2 kHz and it also shown fast response, excellent selectivity, stability, recovery, and reproducibility [218].

**Table 4:** Summary of related SAW sensors for gas detection

**Piezoelectric materials**	**Device frequency (MHz)**	**Sensing layer**	**Target**	**LOD**	**Frequency shift**	**Temp**	**Ref**
Y-Z LiNbO$_{3}$ delay lines	860	ZIF-8	$\textrm{CO}_2$	0.91ppm/vol.	1.11 rad.	RT	[216]
Y-Z LiNbO$_{3}$ delay lines	860	ZIF-8	CH4	16.6 ppm/vol	4.58 rad.	RT	[216]
Y-Z LiNbO$_{3}$delay lines	436	ZIF-8	CH4	7.01 ppm/vol	0.136 rad.	RT	[215]
Y-Z LiNbO$_{3}$delay lines	436	ZIF-8	$\textrm{CO}_2$	0.38ppm/vol	1.396 rad.	RT	[215]
ST-cut quartz.	434.15	Nickel-Alanine- Graphene.	$\textrm{CO}_2$	200 ppm.	2.07 Hz/ppm	RT	[336]
ST-cut quartz.	433.8	AuNR.	VOCs	2.64 ppm	n/g.	RT	[110]
ST-cut quartz.	433	Graphene.	DMMP	10 ppm	−1.4KHz/ppm.	25–100 °C	[337]
ST-cut quartz.	200	GO Nanofilm.	CH3	500 ppb	620 Hz/500 ppb.	RT	[338]
ST-cut quartz.	200	Bi$_{2}$S$_{3}$ nanobelts	NO_2_	1.2 ppm.	2 kHz/10 ppm.	RT	[339]
ST-cut quartz.	200	ZnO-Al$_{2}$O$_{3}$.	H2S	10 ppb.	500 Hz/10 ppb.	RT	[340]
ST-cut quartz.	200	boehmite.	NH3	10 ppm	1540 Hz/10ppm	RT	[341]
ST-cut quartz.	200	CuO-Al2O3	H2S	5 ppb	15 KHz/1ppm.	RT	[342]
ST-cut quartz.	200	Nitrogen doped Diamond.	NH3	100 ppb	0.65 KHz/100ppb.	RT	[343]
ST-cut quartz.	200	SiO$_{2}$, TiO$_{2}$.	NO_2_	16 ppm.	112 KHz/40ppm.	RT	[218]
ST-cut quartz.	200	PbS.	NO_2_	10 ppm.	9.8 KHz/ppm	RT	[217]
ST-cut quartz.	99.5	ZnO.	NO_2_	16 ppm.	112 KHz/40ppm.	RT	[344]
ST-cut quartz.	198.98	CuO.	H2S	500 ppb.	−1200Hz/500 ppb.	RT	[345]

n/g $$=$$ Not Given

Furthermore, the summary of the recent research for the development of the SAW for gas sensor application is presented in Table 4.

### Piezoelectric micromachined ultrasonic transistor (PMUT)

**Fig. 11:**
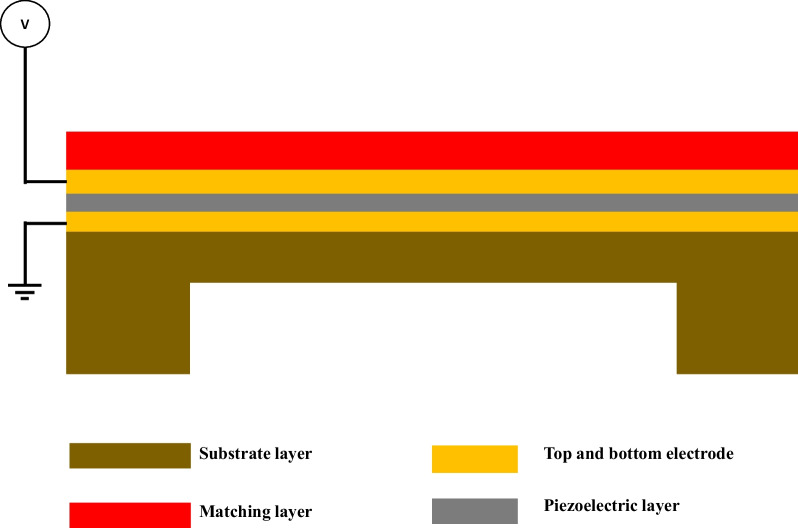
The typical schematic view of the piezoelectric micromachined ultrasonic transducer (PMUT) [227]

The PMUTs are MEMS-based piezoelectric ultrasonic transducers usually used for acoustic imaging of the surrounding environment such as in the medical imaging [219–221], in the automotive [222], fingerprint devices [223–225], fluid density sensing [226] and for gas sensor applications [227]. Although the PMUT and the FBAR are similar in their structure, the PMUT devices are unlike the FBAR solid-based piezoelectric transducers, where the FBAR devices are based on the thickness motion of the piezoelectric plate; however, the PMUTs are based on the bending motion of a thin membrane coupled with a piezoelectric thin film. A typical structure of the PMUT is shown in Fig. 11. Typically, the PMUT has a single piezoelectric layer between the top and bottom electrodes, the electrode should have a specific parameters such as high conductivity, Furthermore, the PMUT can be used as gas sensors by functionalized the top electrode by sensing materials. The sensing materials have significant impact on sensor sensitivity and can influence sensor resonance frequency. PMUT gas sensors offer the ability to overcome some of the problems that other types of gas sensors have, such as power consumption, where the PMUT has the ability to operate by a lower voltage. in addition, the PMUT gas sensors always contained a large top electrode surface which provides enough space for the sensing materials [227].

The working principle of the PMUT gas sensors is mainly determined by the device structure, piezoelectric material thickness, and parameters. For instance, the resonance frequency for a basic PMUT element with one rectangular structure and PZT/Si layered membrane with fully clamped boundaries can be calculated by Eq. (5) by Ref [228, 229]:

5 $$\begin{aligned} f_0=\frac{0.494 t}{w^2} \sqrt{\frac{E}{\rho \left( 1-v^2\right) }\left[ 1+\frac{2}{3}\left( \frac{W}{L}\right) ^2+\left( \frac{W}{L}\right) ^4\right] } \end{aligned}$$

where *L* is the length, *w* is the width, *t* is the thickness, *E* is Young’s modulus, *p* is Poisson’s ratio, and *v* is the density of the material. Equation (5) has clearly highlighted that the resonance frequency of PMUT sensors is primarily governed by the geometry, radius, and thickness of both the piezo-material thin film and the electrodes. Therefore, any modification or defect in these parameters will definitely affect and alter the PMUT resonance frequency. In addition, the operating frequency of the PMUT is typically known to be proportional to Young’s modulus and inversely proportional to the density of the rectangular membrane.

Sun et al. [229] have produced a very sensitive humidity sensor utilizing the PMUT array with a surface functionalized using a graphene oxide thin film. The PMUT sensors have been proposed, fabricated, and tested for humidity sensing where the fabricated sensors showed high sensitivity, good stability, and fast response. Furthermore, Nazemi et al. [227] reported the utilization of the PMUT and CMUT as mass sensors with an extensive investigation of their working principle, device structures and configuration, fabrication processes, critical design parameters, and the resonant frequency changes. The PMUT devices currently are used extensively in ultrasonic imaging production comparing with their application as gas and chemical sensors [222, 230].

### Film bulk acoustic resonator

Over the last two decades, there has been an increased interest in developing and producing high-frequency devices (from sub-GHz to tens of GHz range) such as bulk acoustic waves (BAWs) resonators which have been used as filters [231–236], duplexers [237–239], multiplexers [240, 241], gas sensors [242–245], and chemical biosensors [71, 246, 247]. The film bulk acoustic wave devices are one of the BAW resonators which consisted of a piezoelectric layer usually zinc oxide (ZnO), aluminum nitride (AIN), or lead zirconate titanate (PZT) sandwiched between two metal electrodes to which microwave (RF) signal is applied [245].

The first FBAR device had been disclosed in 1980 by Lakin and Wang [248] and several other groups published similar research during approximately same time [249, 250]. Additionally, FBAR is considered as a development of the previously discovered quartz resonator that has been first reported by Sliker and Roberts in 1967 which consisted of a piezoelectric CdS film on a quartz substrate [251]. However, Lakin and Wang reported a new and unique form of acoustic bulk wave resonator consisted of a thin film of ZnO as a piezoelectric layer which has been sputtered onto a thin silicon membrane supporting structure.

The piezoelectric layer of ZnO is used to excite a longitudinal bulk wave which the wave gets reflected from the membrane and the free surface or the cavity. The authors presented a fabricated device with fundamental resonant frequencies near 500 MHz with a parallel resonant quality factor over 9000 [248]. However, these developed devices in the early stages are operating with less than one GHz resonant frequency and more investigation and development have been done to enhance the sensitivity of the FBAR device through several techniques including the optimization of the resonant frequency and electromechanical coupling coefficient which are considered the most effective method for FBAR sensitivity enhancement and quality factor improvement [119, 252–256].

**Fig. 12:**
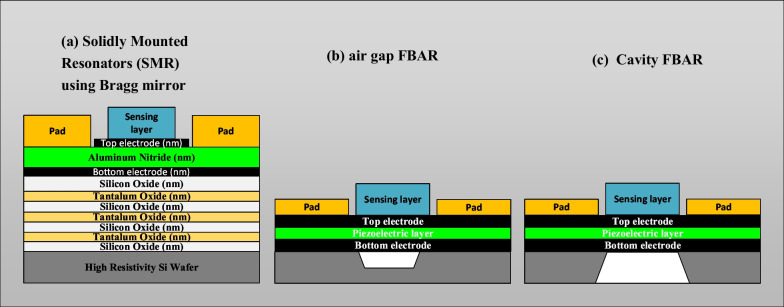
Typical schematic of three different types of FBAR, (**a**) SMR based FBAR [348], (**b**) Air gap based FBAR [349], and (**c**) the Cavity based FBAR using back etch [244, 350]

Additionally, the working mechanism and operation of the FBAR are based on the same principle of the QCM devices; however, FBAR devices have some main differences such as transduction material or piezoelectric material which is sandwiched between the two metallic electrodes and its thickness and size [111, 184, 257]. The quartz crystal material that is being used in QCM has been replaced with thin film piezoelectric material in FBAR devices. Figure 12 schematically presents the cross section of the three different types of FBAR sensors, which are the SMR, air gap-based FBAR and the cavity-based FBAR which has a back-trench structure [245].

Furthermore, FBAR possesses more favored piezoelectric properties including high acoustic velocity [258], high electromechanical coupling coefficient ($k^2$) [259], and low acoustic loss [260]. It has been proven that bringing all these unique properties besides the ultra-thin piezoelectric films such as AIN which is in the thickness of a nanometer, all of these properties can help to produce unique FBAR devices which possess very high resonant frequencies usually from sub-GHz to 10 GHz and high-quality factor [261]. On the other hand, to reflect the acoustic wave, the FBAR active region must be totally isolated from the operational substrate; otherwise, the acoustic wave produced by the piezoelectric film would penetrate the substrate, causing the waves to be lost. As a consequence, there will be no resonance [257].

Therefore, the structure of the FBAR has been developed into two different basic types of device structure. The first structure is the air-cavity resonator which can be further divided into several sub-categories depending on the etching method of the back-trench, such as the air cavity and back-trench which are either etched into or on the substrate [262, 263].

The second FBAR structure is the solidly mounted resonator (SMR) [264] which is made by separating the acoustic resonant wave by using an acoustic Bragg reflector which is consisted of several layers of certain types of materials usually called the Bragg mirror FBAR resonator [265–267]. Both the air cavity and Bragg mirror layer methods have been demonstrated to be effective reflectors and the acoustic waves have been formed between two electrodes known as metallic top and bottom electrodes.

Recently, several methods have been utilized to develop the FBAR structure. One of the various approaches to FBAR structures was created by employing certain materials such as a polymer which has very low acoustic impedance; therefore, it has shown excellent properties to be used as the acoustic reflector; therefore, the FBAR may be manufactured on any solid substrate, such as copper film or glass [268–270].

**Fig. 13:**
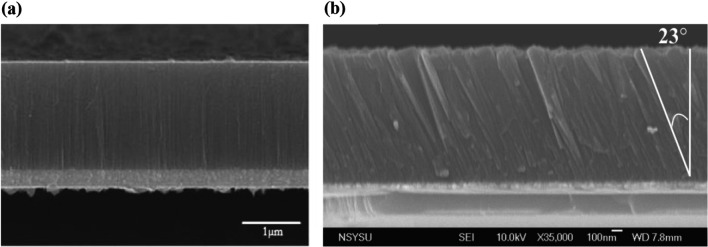
SEM images of the piezoelectric thin film (**a**) cross-sectional view of AIN in the longitudinal mode, produced by [351] (**b**) cross-sectional view of AIN in the f shear mode produced by [274]

In more detail, the FBAR can be operated in two basic resonant modes: The first mode is known as the longitudinal mode, and it generates a longitudinal acoustic wave across the two surfaces of the top and bottom electrodes when an RF signal is applied to both electrodes [271–273]. The second mode is known as the thickness-shear mode, and it occurs when a shear wave is formed between the top and bottom electrodes as a response to the applied alternating voltage. The main differences between these two vibration modes are depending on the c-axis angle of the piezoelectric films. In the fabrication of the FBAR devices with shear mode, the crystal orientation of the piezoelectric material is usually off the c-axis [274], whereas the FBAR with the longitudinal mode, the piezoelectric films are usually fabricated with a crystal orientation that is normal to the film plane or substrate as shown in Fig. 13

The performance of the FBAR with longitudinal and shear mode have been investigated experimentally in the air and liquid environment. In the liquid environment, the shear mode presented high sensitivity and quality factor because the shear waves travel in plane with little damping of resonant waves in liquid, whereas the longitudinal mode waves demonstrated excellent performance such as high sensitivity and high-quality factor in the air environment, however, less responses in the liquid environment [71, 275]. Therefore, the shear mode can work in both dry and liquid environments [115]; however, the longitudinal is only able to work outside liquid conditions. As a result, the shear mode can be utilized in the biosensor application and gas sensors, but the longitudinal mode is only suitable for gas sensors [112, 276, 277].

Furthermore, the behavior of the resonant frequency of the FBAR has been proven theoretically and experimentally in several publications. It is well known that the resonant frequency of the FBAR decreases when additional mass is added to the device’s active area surface. Therefore, in the FBAR gas sensors, the gas adsorption by the sensing layer can be monitored through measuring the changes in the resonant frequency which is affected by the mass changes. Sauerbrey identified the link between increased mass and resonant frequency shift in 1959 [173] as shown in Eq. (6):

6 $$\begin{aligned} \Delta f=-\frac{2 f_{r}^{n}}{A \sqrt{\rho _{q} \mu _{q}}} \Delta m \end{aligned}$$

where $\Delta {f}$ is the frequency change (Hz), $f_{r}$ is the resonant frequency (Hz), $\Delta {m}$ is the mass changes in the surface of the active layer (g), *A* is the piezoelectrically active area ($cm^2$), $\mu _{q}$ is the shear modulus of piezoelectric material (g/cm s2), ${p_{q}}$ is the density of the piezoelectric material (g/cm3), and *n* is number between 1 and 2 applicable for biosensors application. Equation (6) was developed to express the relationship between the additional mass and the responses of the resonant frequency.

In addition, the Sauerbrey equation (6) is dependent on another equation which is used to calculate the frequency resonant as shown in equation (7):

7 $$\begin{aligned} f_{r}=\frac{v_{s}}{2 h} \end{aligned}$$

where *h* is the thickness of the piezoelectric thin film, and $v_{s}$ is the acoustic velocity. Therefore, the resonant frequency is always determined and modified through the thickness of the piezoelectric material. In the FBAR sensors, the thickness of the used piezoelectric thin film is usually in the sub-micrometer to micrometer range, giving resonance frequencies varying between a few hundreds of MHz to 10 GHz and more than in some FBAR with thinner piezoelectric films in the range of nanometers [278]. According to the Sauerbrey equation, the sensitivity of the FBAR resonator is proportional to the device’s resonant frequency and inversely related to the active area of the sensor. From these two parameters, the FBAR is considered more advanced compared with QCM and SAW [59, 184].

Furthermore, The FBAR quality factor is a dimensionless quantity that represents the resonator’s underdamped performance and expressed the correlation between the resonator bandwidth and its center frequency [279, 280]. On other hand, the quality factor is well known and defined as the ratio of the energy stored in the resonator to the energy dissipated for each electromechanical conversion cycle [281, 282], as presented in Eq. (8):

8 $$\begin{aligned} Q=2 \pi \frac{ \text{ Energy } \text{ stored } }{ \text{ Energy } \text{ dissipated } \text{ per } \text{ cycle } } \end{aligned}$$

where the Energy stored is represented the vibration energy stored in the resonator which is divided by the energy of the vibration that dissipated per each cycle. The device with a high-quality factor usually has less energy dissipation per each cycle. In the last few decades, researchers have been investigating the energy loss mechanism in MEMS resonators to enhance the device’s performance. The most relevant loss mechanisms in the piezoelectric MEMS resonators such as the Lame wave resonators are the loss caused by the anchor, interface between the parts loss, thermoelastic damping (TED), material damping, as well as other unknown causes of loss. It has been proven that in the Lame wave resonators, the anchor loss is responsible for the largest proportion of the various energy losses in the MEMS resonators [283–286].

Additionally, the quality factor can also be expressed in the term of resonant frequency to the bandwidth as shown in [287]. The bandwidth is determined by taking the $-3$ dB points difference as expressed in Eq. (9):

9 $$\begin{aligned} Q=\frac{f_{\textrm{r}}}{\Delta f} \end{aligned}$$

where $f_{\textrm{r}}$ is the resonant frequency, and the $\Delta f$ is the differences between the two frequencies before and after the detection processes. The FBAR sensors with a high-quality factor values are gives a more accurate reading in monitoring small frequency shifts. The FBAR with high Q usually has sharper resonant peaks compared with FBAR sensors that have lower Q values. Therefore, the sensitivity of the FBAR sensors is principally determined by both the values of resonant frequency and the quality factor.

Furthermore, the FBAR sensors are basically excited by applying a radio frequency signal on both the top and bottom electrodes of the FBAR sensor; therefore, the device performance is significantly governed by various parameters such as the temperature, piezoelectric materials properties, and electromechanical coupling coefficient ($k^2$) [288]. The electromechanical coupling coefficient is a measurement that is used to express what portion of the applied energy is incorporated or linked into the device [111]. The ($k^2$) can be calculated as expressed in Eq. (10):

10 $$\begin{aligned} k^{2}=e_{31}^{2} /\left( c_{11} \cdot \varepsilon _{33}\right) \end{aligned}$$

where $e_{31}^{2}$ is the electric field, the $c_{11}$ is the elastic constant, and $\varepsilon _{33}$ is the permittivity of the piezoelectric material. It is obvious that the $k^{2}$ depends on the properties of the piezoelectric material used in the device, and the electric field of the device [289]. In addition, there are several other factors that can affect the $k^{2}$ of the FBAR sensors such as the loss in the piezoelectric thin film and the electrode material, thickness, and other properties [118]. The effective electromechanical coupling coefficient $k_{\text{ eff } }^{2}$ is the most common term used for the piezoelectric material assessment. The $k_{\text{ eff } }^{2}$ can be evaluated by Eq. (11):

11 $$\begin{aligned} k_{\text{ eff }}^{2}=\frac{\pi ^{2}}{4}\left( \frac{f_{p}-f_{s}}{f_{p}}\right) ; \text{ or } k_{\text{ eff }}^{2}=\frac{f_{p}^{2}-f_{s}^{2}}{f_{p}^{2}} \end{aligned}$$

where $f_{p}$, $f_{s}$ are the parallel and series resonance frequencies, respectively. The value of this assessment for FBAR sensor is a relatively small value like other acoustic resonators, which is mostly less than 10%.

Furthermore, the quality and performance of the FBAR sensors are significantly affected by the properties and the quality of the piezoelectric thin film material. Various requirements must be considered to fabricate high-quality FBAR sensors including excellent piezoelectric properties with high electromechanical coupling coefficient $k^2$, perfectly organized microstructures such as off-axis orientation with a certain angle for shear mode, easy fabrication process with low cost, etc. [71, 258].

Therefore, the selection of the piezoelectric material is crucial for the development of the FBAR sensors. Currently, there are a variety of piezoelectric materials that have been used for FBAR such as aluminum nitride (AIN), zinc oxide (ZnO), gallium arsenide (GaAs), lead zirconate (PZT), and polyvinylidene fluoride (PVDF). Each of these materials has some strengths and limitations in their properties; for instance, the AIN piezoelectric thin films have shown excellent properties such as high phase velocity, which is suitable for high resonant frequency resonator, easy fabrication process provided by MEMSCAP by PiezoMUMPs technology [245, 290, 291], as well as AIN presented strength and chemical stability, although the production procedure for AIN thin film has relatively small ($k^2$) value compared with ZnO.

In addition, the ZnO piezoelectric material thin film has shown good piezoelectric properties and high $k^2$ compared with AIN film, as well as being highly biocompatible which is believed to be excellent for bioapplications. Likewise, ZnO thin film has been used extensively in various applications during certain times; however, ZnO piezoelectric is hard to be fabricated using modern microelectronic fabrication factories such as MEMSCAP as well as it is not COMS compatible; therefore, these limitations have affected utilizing ZnO thin film in mass production and various application. Furthermore, PZT piezoelectric thin film has shown very high piezoelectric constant and ($k^2$) value, but it has some drawbacks such as lower wave velocity, higher acoustic wave attenuation, and some difficulties in thin film fabrication processes [11, 14, 160, 175].

Additionally, PVDF, SiC, and GaAs piezoelectric thin films have not been used extensively due to some disadvantages such as poor piezoelectric properties and high fabrication cost, and expensive material [112]. In fact, there are some other piezoelectric thin film materials that are being developed for FBAR devices such as gallium nitride (GaN) and barium strontium titanate (BST), particularly for high-frequency devices for communication applications. To culminate with the piezoelectric material that has been used for thin film fabrication for FBAR development, the AIN and ZnO have been strongly considered the most used and useful piezoelectric materials for fabricating FBAR devices, and the choice between both is depending on the applications and fabrication tools accessibility [292–294].

**Fig. 14:**
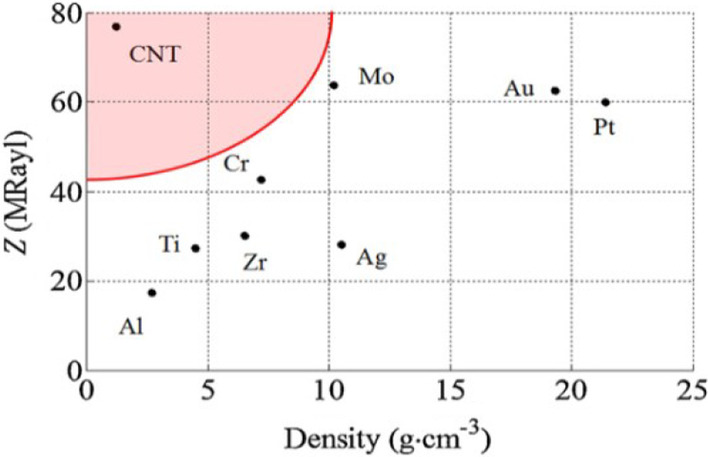
Density and acoustic impedance of commonly used electrode metals, produced by [295]

Furthermore, various methods have been investigated and developed for improving the quality factor, sensitivity, and performance of the FBAR sensors including the choice and development of the piezoelectric thin film material as well as the bottom and top electrode materials, optimizing the device structures, the thickness of the piezoelectric thin film, and the fabrication processes for the FBAR. In addition to the previous explanation for the piezoelectric thin film material properties, the properties of the top and bottom electrodes material are also affecting the performance of the FBAR sensors significantly. The electrode materials must have certain properties to reflect the wave propagation such as high elastic modulus, high acoustic impedance, low mass density, high conductivity, and high acoustic impedance that mismatch with the piezoelectric thin film [295]. In particular, the material with high mass density and low acoustic impedance is not suitable for the electrodes as shown in Fig. 14 which clearly demonstrated the Density and the acoustic impedance of commonly used materials as top and bottom electrode metals. The best material would be located at the top-left corner such as the CNT, Mo, and Cr, these materials have low mass density and high acoustic impedance. Therefore, the most appropriate materials for the FBAR electrode are graphene and CNT which possess low mass density and high acoustic impedance [295]. There are still various materials with unique properties that have yet to be investigated to enhance the properties of the piezoelectric layer and electrodes [295].

Chang et al. [296] have fabricated high working frequency FBAR sensors (4.44 GHz), each individual FBAR sensor was coated with different self-assembled monolayers (SAMs) sensitive layers to establish such E-nose gas sensors. Nine different sensitive materials were used for FBAR surface functionalization, and five different VOCs target were used for the FBAR sensor characterization. The nine functionalized FBARs were tested in a glass chamber and the processes were first started by flushing the device and chamber with pure nitrogen to reach a stable baseline. The FBAR sensors successfully responded once they were exposed to VOCs gas where the sensor resonant frequency decreased due to the gas molecule adsorption reaction. Furthermore, the FBAR sensor showed resonant frequency increasing due to the molecule desorption processes due to the replacement of the VOCs gases with nitrogen. Thus, the FBAR sensors with different SAMs have clearly demonstrated that the adsorption–desorption process is a completely reversible process, which is depending on the monolayer surface modifications. The reversibility process is an extremely important feature in the E-nose and gas sensors application since incomplete desorption will give an unreasonable reading and the sensor will be malfunctioning. The authors investigated and presented the effects of the functional group’s properties on the gas–surface interactions.

Chen et al. [297] have fabricated FBAR and developed a promising strategy to combine the benefits of a microelectromechanical system and the nanostructure of nanofibers for the detection of gases in low concentration. The developed FBARs are working at 4.4 GHz which is performing as a sensitive mass loading platform. Polyethyleneimine nanofibers were prepared and directly deposited by the electrospinning method on the FBAR surface. The adsorption and diffusion of the formaldehyde gas were investigated and the three-dimensional structure of the polyethyleneimine nanofibers presented a large surface area for the detection processes. The resonant frequency of the FBAR presented a downshift due to the ultra-small mass change induced by the formaldehyde molecules’ adsorption onto the amine groups that were provided by the polyethyleneimine surface. The fabricated FBAR sensors demonstrated high sensitivity, excellent selectivity, good reversibility, fast and linear response toward formaldehyde molecules. The sensitivity and detection limit were obtained to be 1.216 kHz/ ppb and 37 ppb, respectively.

Wang et al. [298] have developed a microscale AIN-based film bulk acoustic resonator for formaldehyde vapor detection based on a mass-sensitive mechanism. The authors implement layer-by-layer sensitive coating techniques on the resonator surface utilizing single and multi-walled carbon nanotubes/polyethyleneimine multilayers. The FBAR sensor response was observed after several nanotubes/polyethyleneimine layers were deposited on the surface of the FBAR with an almost linear decrease in the resonant frequency. The reduction in the resonant frequency has approved the mechanism of the mass sensing techniques. Furthermore, the FBAR sensor showed a linear relationship between the concentration of the formaldehyde and the resonant frequency shift. The developed sensitive layer presented a random and porous structure and provided a large surface area which provided enough vacancies for gas adsorption, on the other hand, the FBAR sensor demonstrated excellent selectivity toward the formaldehyde gas molecules thanks to the strong affinity provided by the amine groups in the polyethyleneimine layer, thus the selectivity is always depended on the properties and the modification of the sensitive layer such as surface area, quantity, and quality of the amine or other functional groups that generated by the surface functionalization processes [91]. The authors also demonstrated that the number of the sensitive layers had extremely influenced the adsorption behavior and detection processes. The developed FBAR gas sensor works at an ultra-high resonant frequency of 4.8 GHz and shows good sensitivity in the range of 1.29–1.90 kHz ppb-1 and a limit of detection between 24 and 38 ppb [298]. Furthermore, the authors investigated the influence and the effects of the spraying processes on the sensor performance for formaldehyde detection processes. The sensor response and the reaction between the amine functional groups on the polyethyleneimine surface and the target gas were presented and investigated [299].

Furthermore, Song et al. [300] have also utilized self-assembled polyethyleneimine-modified with single-wall carbon nanotubes as a sensitive coating material in the FBAR for formaldehyde gas detection at room temperature. The fabricated FBAR sensor has been fabricated using 1 $\mu {m}$ AIN piezoelectric thin film which provided ultra-high resonant frequency (4.5 GHz). The FBAR has demonstrated the ability to detect small mass changes in the sensitive layer on the top surface of the device which is induced by the adsorption of the target gas molecules. The reversibility and selectivity have been demonstrated by the proposed FBAR sensor. Furthermore, the sensitivity and frequency response of the sensor were significantly improved by increasing the area covered by the carbon nanotubes on the sensor surface due to the excellent properties of the carbon nanotubes such as large surface area, and good adsorption properties. The fabricated FBAR sensor has been tested for formaldehyde molecules detection and the sensor presented a linear response toward the targeted gas in the range of 50–400 ppb with a 24 ppb limit of detection. Therefore, these results prove that the FBAR sensor is a promising device to be used for portable and convenient detection of indoor air pollution at room temperature.

Furthermore, Jilong Ma et al. [301] developed another type of FBAR using a ZnO piezoelectric thin film layer instead of AIN. The prepared FBAR sensors have been used for formaldehyde gas detection. Multi-walled carbon nanotubes/polyethyleneimine bilayer was developed as a sensitive layer for the detection processes and was self-assembled on the resonator surface. The fabricated FBAR sensors have been developed using the Bragg reflector technique through ${\textrm{SiO}}_{2}/{W}$ layers. The sensitivity and selectivity of the FBAR sensors toward formaldehyde molecules have been enhanced by the amine functional groups on the polyethyleneimine. The authors presented a reversible nucleophilic addition reaction between the formaldehyde gas molecules and the amine functional groups that available on the polyethyleneimine surface; thus, the multi-walled carbon nanotubes/polyethyleneimine sensitive layer demonstrated excellent adsorption toward the formaldehyde gas. Additionally, the developed FBAR sensor with a high working frequency of 3.1 GHz has presented high and excellent mass sensitivity and ultra-small mass change detection with a linear relationship between the increase in the formaldehyde gas concentration and the resonant frequency downshift. This FBAR sensor has shown excellent results for formaldehyde gas concentration in the range of 50-400 ppb at room temperature.

In addition, Zeng et al. [302] have developed single film bulk acoustic wave resonator for the detection and discrimination of volatile organic compounds (VOCs) utilizing highly sensitive coated material which consisted of 20-bilayer self-assembled poly (sodium 4-styrene sulfonate)/poly (diallyl dimethyl ammonium chloride) thin film. The authors have conducted proof-of-concept validation processes for the fabricated FBAR by exposing the device to six different VOC vapors at six different gas partial pressures. The device was successfully detecting the target gases in real time with static and dynamic detection. The FBAR frequency shifts and impedance responses were measured and evaluated using several analysis techniques, which present that all types of exposed gases can be detected, classified, and distinguished with an accuracy of more than 97$\%$. The FBAR sensor has been designed to be working with a resonant frequency of 2.45 GHz which provides excellent sensitivity and a low limit of detection. This research investigated the effects of temperature modulation on the adsorption and desorption of different VOCs. Furthermore, the authors presented the capability of the FBAR sensor to discriminate different VOCs by using measuring the frequencies and impedance responses under different temperatures. This type of FBAR gas sensor demonstrated simple mass-based electronic detection tools with ultrasensitive capability, low cost, small size, and stable working temperature with minimizing the affecting of ambient temperature fluctuation. These unique features give the FBAR potential to be used and integrated into small electronic circuits such as mobile phones.

Liu et al. [280] have developed a high-performance film acoustic resonator for humidity mentoring. The published paper presented the design, simulation, fabrication, and characterization of the FBAR sensor for humidity detection. Polyimide film was employed to be used as a humidity-sensing layer and to provide structural support for the device. The FBAR sensor was tested with and without the polyimide film and it has shown that the sensor with thin film coating has 39 times higher response than the sensor without any sensing layer, where the sensitivity reaches + 67.3 kHz$\%$RH between 15$\%$RH and 85$\%$RH. The fabricated FBAR sensor was fixed on a printed circuit board (PCB) and bonded using gold wire. The S-parameters of the FBAR sensor has been measured and characterized using a network analyzer. Furthermore, all the characterization processes for the humidity response measurement were carried out using a humidity generator (MODEL 2000) in the range from 15$\%$RH to 85$\%$RH at room 25 $^{\circ }$C. However, the FBAR humidity sensor is always sensitive to the temperature; therefore, the effects of the temperature on the sensor response have been characterized in the range of $-50 ^{\circ }$C to +50 $^{\circ }$C using another chamber called a high–low temperature alternating test chamber.

In addition, Yan et al. [303, 304] have utilized metal–organic frameworks (MOFs) sensitive material with high surface area (HKUST-1) in film bulk acoustic resonators to enhance the device performance. Since the sensor parameters such as sensitivity, selectivity, and the stability are mainly dependent on the properties of the prepared nanomaterials, the authors investigated various hybrids between organic and inorganic which significantly enhanced the sensor’s performance. The fabricated FBAR sensor has a working resonant frequency of around 2.4 GHz and it has been used to detect several types of VOCs and water molecules. The sorptive MOF thin film was prepared through a layer-by-layer strategy.

Zhang et al. [305] have designed and fabricated FBAR gas sensors for relative humidity (RH) and ethanol detection. The fabricated FBAR was driven by a Colpitts oscillator which provides and supplies a frequency signal for the detection application. The device is fabricated from a multilayer structure, consisting of two electrodes sandwiching the ZnO piezoelectric layer. In this FBAR sensor, the ZnO has been used as piezoelectric material and sensitive layer due to the unique properties of the ZnO such as the strong physical adsorption and sensitive chemical adsorption between the crystalline ZnO and various chemicals such as ethanol, hydrogen, ozone, and water. The authors have introduced new micro through holes with a size of 10 $\mu {m}$ * 10 $\mu {m}$ within the top electrode to enlarge the reaction between the ZnO and the targeted gas molecules which will enhance the FBAR sensitivity. The effects of the micro through holes have been demonstrated experimentally where the sensitivity has been enhanced by around 3.2 times higher compared to the FBAR sensor which has a full solid top electrode. Additionally, Agilent Network Analyzer was used to determine the sensor impedance characteristics, In addition, a CXA signal analyzer and a mixed signal oscilloscope were utilized to identify the sensor’s output signals. A Moisture Generator regulated the temperature and humidity, and the detection was conducted in a static condition. The sensor was operated with a voltage supply of 3 V and the sensor’s output signal was detected at a power of $-2.6$ dBm and phase noise $-90$ dBc/Hz@ 100kHz. The sensor was used for humidity detection in the range from 25 to 88$\%$ at room temperature and the resonant frequency shift was 733 kHz.

In addition, the utilization of FBAR as a gas sensor has attracted researchers, and they still investigating the feasibility of the FBAR being used as a portable gas sensor for indoor applications. Hoffmann et al. [306] have fabricated solidly mounted FBAR with ZnO (900 nm) piezoelectric material and a working resonant frequency of around 800 MHz for humidity and carbon dioxide detection at room temperature. Since the FBAR resonant frequency is mainly dependent on the piezoelectric material thickness and acoustic velocity, the ZnO piezoelectric material has less acoustic velocity compared with the AIN materials; therefore, the resonant frequency in this FBAR is less than 1 GHz. The authors investigated two different sensitive materials including polyaminosiloxane and ethyl cellulose to be used for FBAR surface functionalization. The FBAR response for ${\textrm{CO}}_{2}$ has been demonstrated with a resolution of 50 ppm in the range of 400 and 1000 ppm. The density of the adsorption layer and the acoustic velocity of the device have been expressed by using the Mason model and the correlation between the changes in these parameters and the effect in the resonant frequency has been highlighted.

**Fig. 15:**
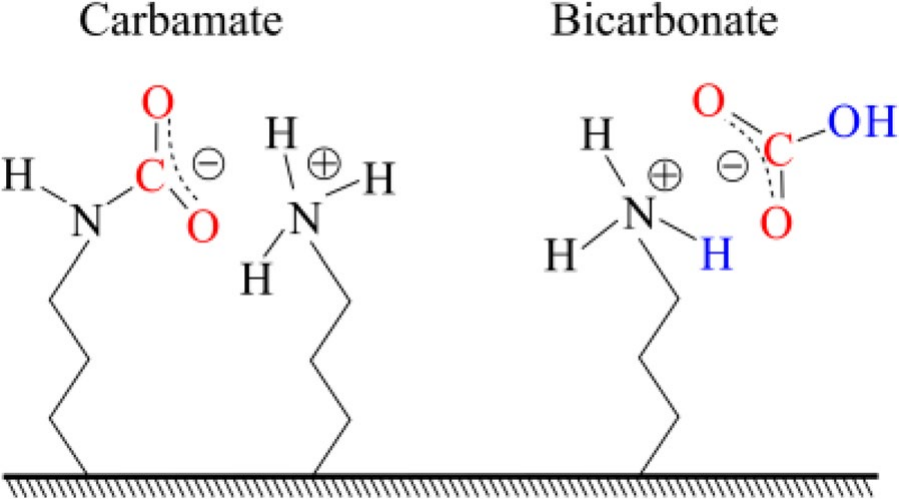
The two most common interactions of $\text{CO}_2$ with amines, where the carbamate needs two amine groups in the vicinity, whereas bicarbonate utilizes water as a free base [306]

Furthermore, the FBAR has been coated with functional groups for ${\textrm{CO}}_{2}$ detection, in fact, there are various types of functional groups that have been used for ${\textrm{CO}}_{2}$ adsorption as summarized by Choi et al. [307]. However, amine-based organic compounds are considered one of the most promising sensitive layers for ${\textrm{CO}}_{2}$ detection application. It has been known that amines can bind with carbon dioxide through acid-based reactions. Furthermore, the reaction between the carbon dioxide and the amine is also depended on the atmosphere such as in an anhydrous atmosphere, there must be two moles of amine for one mole of ${\textrm{CO}}_{2}$ to bind in the form of carbamate as shown in Fig. 15 in addition, in hydrous atmosphere, water molecules can act as an additional free base, thus only one amine group is necessary to be bound with ${\textrm{CO}}_{2}$, and this reaction will form bicarbonate species [307–313].

Furthermore, the film bulk acoustic resonator has attracted researchers’ attention for its promising features, especially in high-frequency applications. The traditional radio frequency filters cannot meet the demands of high-frequency application, integration, and miniaturization features. Therefore, there is a new horizon that appears for FBAR in the high-frequency application. Additionally, there are some scholars extensively investigating the capability of the FBAR for several applications such as communication filters [314–318] and chemical detection [257, 319–321].

**Table 5:** Summary of related FBAR sensors for gas detection

Piezoelectric materials	Device frequency (MHz)	Sensing layer	Target	Limit of detection	Frequency shift	Temp	Year	Ref
AIN	4.8 GHz	CNT/polyethyleneimine	Formaldehyde	24–38 ppb	1.29–1.9 kHz/ppb	RT	2018	[298]
AIN	4.5 GHz	SWCNT/polyethyleneimine	Formaldehyde	82–143 ppb	0.412–0.301 kHz/ppb	RT	2018	[300]
AIN	4.44 GHz	Nine-SAM	VOCs	N/G	n/g	RT	2016	[296]
AIN	4.4 GHz	Polyethyleneimine nanofibers	Formaldehyde	37 ppb.	1.216 kHz/ppb	RT	2018	[297]
ZnO	3.1 GHz	MWCNT/polyethyleneimine	Formaldehyde	50–400 ppb	1.4–2.13 kHz/ppb	RT	2018	[301]
AIN	2.45 GHz	Hybrid thin film	VOCs	40–180 ppb	0.05 kHz/ppm	RT	2019	[302]
AIN	2.4 GHz	MOFs	VOCs	1 ppm	0.28–1.27 kHz/ppm	RT	2020	[303, 304]
ZnO	1.15 GHz	Polyimide	Humidity	15–85%	67.3 kHz/%RH	RT	2020	[280]
ZnO	800 MHz	Ethyl cellulose, poly-siloxanes	CO$_{2}$.	50 ppm	N/G	RT	2017	[306]
ZnO	N/G GHz	ZnO	Humidity, ethanol	25–88%	733 kHz/%RH	RT	2017	[305]

*N/G* Not given

In addition, the researchers are still investigating the FBAR parameters for enhancing the device performance such as the quality factor, coupling coefficient, electromechanical coupling, device geometry, and the figure of merit [261, 268, 288, 322–326]. For instance, Johar et al. [327] have optimized FBAR for toluene gas detection. The authors reported an optimized design of a flexible film bulk acoustic resonator with polyethylene terephthalate as a flexible substrate. Furthermore, the paper investigated the performance of different piezoelectric materials such as aluminum nitride, zinc oxide, and zirconate titanate with a polydimethylsiloxane (PDMS) sensitive layer coated on the top electrode for toluene gas detection. Furthermore, the Bragg reflector stages and the effect of the PDMS layer thickness on the performance of the sensor were presented using finite element modeling and the optimal configuration was obtained using Taguchi DoE and ANOVA techniques. The results for the optimized structure have been presented for the coupling coefficient, quality factor, and figure of merit which are 23.7874$\%$, 991, and 235, respectively. Table 5 summarizes the related FBAR sensors that have been developed for gas detection applications.

### Summary of common piezoelectric-based sensors for gas detection

**Table 6:** Summary of common piezoelectric-based sensors for gases detection

Device type	Piezoelectric material	Device frequency (GHz)	Sensing material	Targeted gas	LOD (ppm)	Frequency shift (Hz/ppm)	Response time (S)	Recovery time (S)	Temp	Year	Refs
QCM	AT-CUT	0.005	Polymer/GNR	NH$_{3}$	70	106	1800	n/g	RT	2021	[169]
QCM	AT-CUT	0.009	ZIF	CO$_{2}$	n/g	n/g	n/g	n/g	RT	2018	[333]
QCM	AT-CUT	0.009	(ZIF-8)	CO$_{2}$	10,00,000	217	10	n/g	RT	2018	[215]
QCM	AT-CUT	0.01	rGO	CO$_{2}$	50–500	50	26	10	RT	2021	[176]
QCM	AT-CUT	0.01	MOF	CO$_{2}$	400–500	n/g	n/g	n/g	RT	2018	[179]
QCM	AT-CUT	0.025	PAAM/MWCNTs	Formaldehyde	0.5	3	80	100	RT	2021	[331]
SAW	ST-cut quartz	0.2	SiO$_{2}$, TiO$_{2}$	NO$_{2}$	16	112 KHz/40ppm	80	50	RT	2017	[218]
SAW	ST-cut quartz	0.4341	Nickel–Graphene	CO$_{2}$	200	2.07	–	–	RT	2015	[336]
SAW	ST-cut quartz	0.2	GO Nanofilm	CH$_{3}$	500	620 Hz/500ppb	100	500	RT	2019	[338]
SAW	ST-cut quartz	0.2	Boehmite.	NH$_{3}$	10	1540 Hz/10ppm	n/g	n/g	RT	2019	[341]
SAW	Y-Z LiNbO$_{3}$	0.436	ZIF-8	CO$_{2}$	0.38	1.396 rad	n/g	n/g	RT	2018	[215]
SAW	Y-Z LiNbO$_{3}$	0.86	ZIF-8	CO$_{2}$	0.91	1.11 rad	n/g	n/g	RT	2020	[216]
FBAR	ZnO	0.8	Ethyl cellulose	CO$_{2}$	50 ppm	n/g	n/g	n/g	RT	2017	[306]
FBAR	AIN	2.4	MOFs	VOCs	1 ppm	0.28–1.27 KHz/ppm	n/g	n/g	RT	2020	[303, 304]
FBAR	AIN	2.45	Hybrid thin film	VOCs	40–180 ppb	0.05 KHz/ppm	n/g	n/g	RT	2019	[302]
FBAR	ZnO	3.1	MWCNT -hybrid	Formaldehyde	50–400 ppb	1.4-2.13 KHz/ppb	–	-	RT	2018	[301]
FBAR	AIN	4.4	polyethyleneimine nanofibers	Formaldehyde	37	1.216 KHz/ppb	25	60	RT	2018	[297]
FBAR	AIN	4.8	“Polyethyleneimine /CNT”	Formaldehyde	24–38 ppb	1.29-1.9 KHz/ppb	57	59	RT	2018	[298]

n/g = not given

The piezoelectric-based gas sensors have been summarized in this section as presented in Table 6. The piezoelectric sensors that work based on the QCM technology usually have less resonant frequency compared with the FBAR sensors; therefore, the QCM sensors are not provided highly sensitive features and its bulk quartz layer is not compatible with CMOS fabrication technology. As a result, the QCM is not supporting the future trends of sensors integration and monolithic approaches. Furthermore, the piezoelectric gas sensors based on the SAW technology have a working resonant frequency of less than 1 GHz, and the acoustic wave is propagating on the surface of the device compared with the FBAR in which the acoustic wave is propagating inside the bulk piezoelectric layer; therefore, the SAW sensors are provided low sensitivity compared with the FBAR sensor. In addition, from the literature, the PMUT sensors are mainly used for medical imaging and in fingerprint application rather than the gas sensor application. Therefore, the FBAR technology seems to provide a unique platform for gas sensing applications, especially since it has the feasibility to be fabricated with CMOS fabrication compatibility and monolithically.

## Summary

The present work reviewed the scientific literature that addressed the piezoelectric-based MEMS gas sensors, starting from the historical background of the piezoelectric sensors and including the development of each piezoelectric device, their design and simulation, experiments, and sensing capabilities. Several piezoelectric transducers have experimented for detecting small trace gas molecules. The most commonly developed piezoelectric-based MEMS have been investigated in detail, such as the piezoelectric microcantilever, surface acoustic wave, quartz crystal microbalance, piezoelectric micromachined ultrasonic transducer, and the film bulk acoustic wave resonators. In particular, the papers that quantitatively demonstrated the capability of the piezoelectric devices through simulation, fabrication, and verification have been analyzed comprehensively. Overall, these piezoelectric-based MEMS sensors demonstrated unique properties to be used as gas sensors which have the capability of detecting trace gas molecules and have the feasibility to be fabricated with CMOS technology to be built-in sensors.

## Data Availability

The data reported in this research are available from the corresponding author upon request.

## Abbreviations

MEMS: Microelectromechanical system
Piezo-MEMS: Piezoelectric microelectromechanical system
QCM: Quartz crystal microbalance
SAW: Surface acoustic waves
PMUT: Piezoelectric micromachined ultrasonic transducer
FBAR: Film bulk acoustic wave resonators
FPAR: Film plate acoustic resonators
LE: Lateral extensional mode
LFE: Lateral field excitation
Flex: Flexural mode
TE: Thickness extensional mode
CMR: Contour-mode resonator
TSM: Thickness shear mode
APW: Acoustic plate wave
BAW: Bulk acoustic wave
SH-SAW: Shear horizontal acoustic wave
AIN: Aluminum nitride
